# *E Unibus Plurum*: Genomic Analysis of an Experimentally Evolved Polymorphism in *Escherichia coli*

**DOI:** 10.1371/journal.pgen.1000713

**Published:** 2009-11-06

**Authors:** Margie A. Kinnersley, William E. Holben, Frank Rosenzweig

**Affiliations:** Division of Biological Sciences, University of Montana, Missoula, Montana, United States of America; University of Toronto, Canada

## Abstract

Microbial populations founded by a single clone and propagated under resource limitation can become polymorphic. We sought to elucidate genetic mechanisms whereby a polymorphism evolved in *Escherichia coli* under glucose limitation and persisted because of cross-feeding among multiple adaptive clones. Apart from a 29 kb deletion in the dominant clone, no large-scale genomic changes distinguished evolved clones from their common ancestor. Using transcriptional profiling on co-evolved clones cultured separately under glucose-limitation we identified 180 genes significantly altered in expression relative to the common ancestor grown under similar conditions. Ninety of these were similarly expressed in all clones, and many of the genes affected (e.g., *mglBAC*, *mglD*, and *lamB*) are in operons coordinately regulated by CRP and/or rpoS. While the remaining significant expression differences were clone-specific, 93% were exhibited by the majority clone, many of which are controlled by global regulators, CRP and CpxR. When transcriptional profiling was performed on adaptive clones cultured together, many expression differences that distinguished the majority clone cultured in isolation were absent, suggesting that CpxR may be activated by overflow metabolites removed by cross-feeding strains in co-culture. Relative to their common ancestor, shared expression differences among adaptive clones were partly attributable to early-arising shared mutations in the *trans*-acting global regulator, rpoS, and the *cis*-acting regulator, *mglO*. Gene expression differences that distinguished clones may in part be explained by mutations in *trans*-acting regulators *malT* and *glpK*, and in *cis*-acting sequences of *acs*. In the founder, a *cis*-regulatory mutation in *acs* (acetyl CoA synthetase) and a structural mutation in *glpR* (glycerol-3-phosphate repressor) likely favored evolution of specialists that thrive on overflow metabolites. Later-arising mutations that led to specialization emphasize the importance of compensatory rather than gain-of-function mutations in this system. Taken together, these findings underscore the importance of regulatory change, founder genotype, and the biotic environment in the adaptive evolution of microbes.

## Authors Summary

Experimental evolution of asexual species has shown that multiple genotypes can arise from a single ancestor and stably coexist (*e unibus plurum*). Although facilitated by environmental heterogeneity, this phenomenon also occurs in simple, homogeneous environments provisioned with a single limiting nutrient. We sought to discover genetic mechanisms that enabled an *E. coli* population founded by a single clone to become an interacting community composed of multiple clones. The founder of this population contained mutations that impair regulation of acetate and glycerol metabolism and likely favored the evolution of cross-feeding. Adaptive clones share *cis*- and *trans*-regulatory mutations shown elsewhere to enhance fitness under glucose limitation. Certain mutations that distinguish adaptive clones and underlie evolution of specialists were compensatory rather than gain-of-function, and all that we detected resulted in gene expression changes rather than protein structure changes. Evolved clones exhibited both common and clone-specific gene expression changes relative to their common ancestor; the pattern of gene expression in the dominant clone cultured alone differed from the pattern observed when it was cultured with variants feeding on its overflow metabolites. These findings illuminate the roles played by founder genotype, differential gene regulation, and the biotic environment in the adaptive evolution of bacteria.

## Introduction

Evolutionary biologists have long sought to understand mechanistically how adaptive genetic variation arises and persists. Experimental studies using model organisms such as *Drosophila* [1]–[3] and *C. elegans* [4]–[6] transformed the search for such mechanisms from a retrospective to a prospective endeavor. But, long generation times, sexual recombination and practical limits on lab population size make higher eukaryotes imperfectly suited to study the tempo, trajectory and mechanisms by which evolution occurs in asexual species and in the somatic cells of sexual organisms. There, new genetic variation is limited by the rate of mutation supply and, in bacteria, also by the incidence of horizontal gene transfer. Fortunately, evolution in asexual species and cells can be studied using microbial models [7],[8]. Early microbial studies helped lead to two generalizations concerning the emergence and persistence of genetic variation in large, asexual populations. First, over *ecological time* and in the absence of spatial structure and differential predation, competition for the same limiting resource selects for one fittest variant, an insight that came to be known as the “competitive exclusion principle” [9],[10]. Second, over *evolutionary time* variation arising by mutation is subject to “periodic selection” leading to a succession of genotypes each more fit than its immediate predecessor [11]–[13]. These generalizations led to the expectation that large, clonal populations evolving under resource limitation should exhibit limited genetic variation.

Experimental evidence now suggests otherwise. Multiple genotypes which arise from a single ancestral clone can coexist over evolutionary time; in other words, out of one comes many (*e unum pluribus*). This phenomenon has been documented in spatially and temporally unstructured chemostats [14],[15], in temporally-structured batch cultures [16]–[20], and in spatially-structured microcosms [21]. In each setting, the emergence and persistence of polymorphism in the absence of sexual recombination seems to require that cohabitants exploit alternative ecological opportunities (i.e., unoccupied niche space), and/or accept trade-offs between being a specialist and a generalist (as reviewed in [22], also see [23]. In serial dilution batch culture multiple growth parameters can come under selection [reviewed in 24]. Different clones may arise that have reduced lag time, increased maximum specific growth rate, or enhanced capacity to survive at high cell densities in the presence of low nutrients. Periodic changes in population density and nutrient levels may bring balancing selection to bear on these different phenotypes, especially if antagonistic pleiotropy precludes evolution of one fittest genotype having all of these advantageous traits. In spatially structured environments selection may favor mutants better adapted to particular regions or better able to colonize microhabitats formed at the boundaries between such regions. In continuous nutrient-limited environments (e.g., chemostats), theory [25]–[27] predicts that selection will favor clones better able to scavenge the limiting resource or more efficiently convert that resource to progeny. Ultimately, the outcome of the “evolutionary play” in any of these “ecological theaters” will depend on founder genotype, the complexity of genetic pathways which lead to different adaptive strategies, as well as the propensity of key steps along those pathways to undergo mutation and to act pleiotropically.

Only recently have we begun to discover genetic mechanisms that explain how balanced polymorphisms arise and persist in large, asexual populations. In serial batch culture, differences in the activity of the global regulator RpoS help explain co-existence of two *E. coli* isolates with different propensities to survive extended stationary phase [28]; the precise genetic basis for these activity differences, however, remains obscure. In a spatially structured microcosm founded by a single clone of *Pseudomonas fluorescens*, a methylesterase structural mutant arose and persisted because the resulting change in exopolysaccharide production enabled the mutant to colonize the air-broth interface [29]. Finally, in glucose-limited chemostats polymorphic *E. coli* populations repeatedly evolved, in part owing to local regulatory mutations that affect expression of a single operon (*acs-actP-yjcH*) [30]. When adaptive clones from one such population were grown in monoculture, strain-specific differences in ca. 20% of identifiable proteins expressed suggested the presence of other mutations with highly pleiotropic effects [31]. Thus, regardless of experimental system, uncertainty remains as to whether either regulatory or structural mutations consistently deliver greater fitness increments, which category of mutation better explains the maintenance of diversity, and whether one type is more likely to precede the other in an evolutionary sequence leading to balanced polymorphism.

Theoretical considerations have led some to argue that the major phenotypic changes which underlie adaptive radiation are more likely due to regulatory than to structural mutations [32],[33 and refs. therein]. This argument is based on the perception that changes in coding sequences are more likely to have large pleiotropic effects than changes in the expression of those sequences, in particular changes that arise from the mutation of *cis*-regulatory elements affecting single genes. In effect, this type of regulatory mutation enables selection more easily to “tinker” (*sensu* Jacob, 1977), as it provides a mechanism to alter functionality in one process while still preserving the role of pleiotropic genes in others [34]. Also, and this fact too often goes unappreciated, a discrete *cis*-regulatory mutation preserves the capacity to restore the ancestral pattern of expression via compensatory or back-mutations. The proposition that regulatory mutations play a greater role in adaptive diversification has been criticized on empirical and theoretical grounds by Hoekstra and Coyne who point out the vastly greater number of examples where adaptation is attributable to structural rather regulatory mutations, as well as the facts that *cis*-acting elements offer much smaller targets for mutation than ORFs, and that in many species pleiotropic effects arising from structural mutations may be buffered by gene duplication [35].

While the “*cis*-regulatory hypothesis” is based largely on a consideration of multicellular eukaryotes, tends to be focused on events that transpire during plant and animal development, and requires what we view to be an artificial distinction between physiological and morphological adaptation, it nevertheless provides a useful framework in which to make predictions about how adaptive diversification might occur in ‘simpler’ species. In bacteria, both types of mutations can be evolutionarily significant. Structural mutations in the HopZ family of Type III secreted effector (T3SE) proteins play a major role in pathoadaptation by *Pseudomonas syringae* to its plant hosts [36]; likewise, T3SE mutations underlie host immune suppression by *Yersinia* and *Xanthomonas* [37]. On the other hand, pathoadaptation leading to an intracellular lifestyle in *Samonella enterica* results from a *cis*-regulatory mutation, specifically, acquisition of a binding site for pathogenicity island-2 regulator SsrB [38].

We sought to address the issue of structural versus regulatory change in *Escherichia coli* by investigating an experimental population first described by Helling et al. [14]. This population was founded by a single clone and evolved in an aerobic, glucose-limited chemostat at constant dilution rate (D = 0.2 h^−1^) and constant temperature (30°C). Helling et al. inferred from fluctuations in a neutral marker that adaptive mutations occurred about once every 100 generations. At the time they concluded their experiment (765 generations) they could distinguish four strains on the basis of colony size and ampicillin sensitivity (see Table 1). Three of these phenotypes were shown to stably co-exist in reconstruction experiments, wherein the majority clone strain, CV103, was followed in rank order of abundance by CV116 and CV101 [15]. Each strain exhibited a characteristic pattern of protein expression, as determined by 2D protein gel electrophoresis, when grown by itself in glucose-limited chemostats; as a group, evolved clones significantly differed from their common ancestor at ∼160 expressed proteins of ∼700 that could be resolved [31].

**Table 1:** Bacterial strains.

Strain	Relevant Characteristics^2^	Specific growth rate (hr^−1^) 2	Relative growth yield^3^	Rate of glucose uptake (µmol αMG/min/gm)^3^	Equilibrium [glucose] (nmol/mL)^3^	Equilibrium [acetate] (nmol/mL)^3^
**RH201** 1	CGSC 5346 F- *thi 1leu6 thiI lacY1 tonA21 supE44 hss1 glpR200*					
**JA104**	Derivative of RH 201 F- *thi 1 lacY1 araD139gdh supE44 hss1*; lysogenic for λ					
**JA122**	As JA104 but contains plasmid pBR322Δ5	0.44±0.01	1.14±0.02	1.19±0.09	1.84±0.48	194±20
**CV101**	Derivative of JA122; isolated after 773 generations, Amp^R^	0.50±0.02	1.11±0.02	1.66±0.06	0.88±0.31	0±0
**CV103**	As CV101 but independent isolate which forms small colonies on T, Amp^R^	0.40±0.01	0.81±0.04	2.46±0.16	0.07±0.03	252±70
**CV115**	Derivative of JA122, isolated after 773 generations, lacks plasmid	0.55±0.02	1.11±0.02	ND	ND	ND
**CV116**	As CV115 but forms small colonies on TA	0.60±0.01	1.20±0.03	1.61±0.11	0.19±0.05	40±25

^1^Ref. [113].

^2^Data from [14], Table 1.

^3^Data from [15], Table 2.

Relative to their common ancestor, all evolved clones showed enhanced uptake of the glucose analogue ^14^C-α-methylglucoside (αMG), and CV103 accumulated significantly more α–MG than any other clone [14], even though its yield was less than the other adaptive clones. Equilibrium glucose concentration (the amount detectable in a culture of actively dividing cells at steady state) was an order of magnitude less in CV103 than in CV101 chemostats and less than half that observed in CV116 (see Table 1). Unlike CV101 and CV116, however, CV103 left metabolizable carbon in the chemostat, effectively creating niches conducive to the evolution of cross-feeding. The other strains filled those niches, efficiently scavenging overflow metabolites below detection limit [15]. Acetate-scavenging strains were subsequently observed in 6 out of 12 independent evolutionary populations founded by cells of similar genetic background grown under similar conditions [39].

The Helling and Adams population is a classic example of how adaptive evolution occurs in the context of niche diversification. Because the approximate number of fixed adaptive mutations is few and the number of significant changes in protein expression is many, this population is well-suited for identifying mutations that exert large effects, and determining whether those mutations occur at loci that encode enzymes in metabolism, at *trans*-acting loci that encode proteins which regulate expression of multiple enzymes, or at *cis*-acting sequences that control how activators and repressors act on single genes. We tested the hypothesis that enhanced uptake and assimilation of the primary resource, glucose, results from one (or few) early-arising mutations in *trans*-acting global regulators, and that specialization on secondary resources arises from later-arising mutations in key structural loci or in their *cis*-acting sequences. Lastly, we anticipated that in comparing the consortium's expression profile to that of individual members grown in monoculture, we would discover emergent properties of the system not apparent using a purely reductionist approach.

Transcriptional profiling of evolved strains in monoculture reveals ∼180 genes significantly altered in expression. Many shared increases and decreases are attributable to shared mutations in *rpoS* and the maltose operon operator *mglO*. Expression differences that distinguish isolates occur mainly in the majority clone, CV103. Many of these genes are regulated (or are predicted to be regulated) by cAMP receptor protein (CRP) and/or the global stress regulator CpxR. The “community” expression profile is strikingly similar to the monoculture profiles of the three sub-dominant clones, suggesting that biochemical interactions among clones alter CRP-CpxR regulation. We identified in the founder regulatory mutations in genes required for acetate and glycerol catabolism that likely predispose this system to the evolution of cross-feeding. Among adaptive clones, we found shared mutations in *rpoS* and *mglO*, and mutations that distinguish clones from one another at _p_ *acs*, *malT* and *glpK*. Taken together, our results suggest that both *cis*- and *trans-*regulatory changes underlie adaptive diversification in a simple, unstructured, resource-limited environment, and that founder genotype and chemical interactions among clones not only facilitate co-evolution, but also strongly impact their respective patterns of gene expression.

## Results

### Bacterial strains

Table 1 summarizes previously published phenotypic data on the Helling et al. strains which are germane to interpretation of the expression and sequencing Results presented below [14],[15].

### Genomic characterization

**Figure 1:**
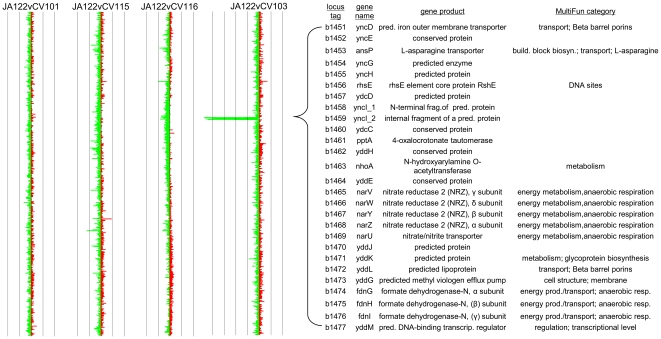
Array Comparative Genomic Hybridization (a-CGH) of each adaptive clone *versus* their common ancestor, JA122. CV103 has sustained an approximately 29 Kb deletion relative to JA122 comprising a total of 27 genes of either unknown function or involved in transcription, arginine biosynthesis, anaerobic respiration, nitrogen metabolism and glycoprotein biosynthesis. Cy-5 labeled genomic DNA from each evolvant (red bars) was hybridized against Cy-3 labeled genomic DNA from JA122 (green bars). The log_2_ ratio of hybridization intensities is depicted along a linear map of the *E. coli* K-12 MG1655 chromosome with genes closest to the origin at the top. Grey lines denote a 2-fold difference in target hybridization. The deleted portion of the CV103 chromosome shown as an excess of hybridization in the reference channel encompasses the 27 genes detailed in the table to the right.

To assess the level of large-scale genetic variation between the ancestor and the evolved clones, we performed rep-PCR fingerprinting and array-CGH. BoxA1R rep-PCR fingerprints were indistinguishable (see Figure S1). However, a-CGH revealed a deletion of ∼30 Kb in the majority clone, CV103 (Figure 1). A total of 27 genes were lost by the deletion, 12 of which have no known function. Of the remaining 15, 3 have a predicted function based on homology to previously characterized genes and 12 are involved in a variety of cellular processes including transcription, arginine biosynthesis, anaerobic respiration, nitrogen metabolism and glycoprotein biosynthesis.

### Adaptive clones display common patterns of gene expression in chemostat monoculture

We used DNA microarrays to assess global transcriptional patterns of individual strains in glucose-limited chemostats. Evolved clones were grown to steady state (∼14 generations) under conditions identical to those under which they evolved (D = 0.2 h^−1^, 30°C). In each case, steady state transcript levels were estimated in relation to the ancestral strain JA122 grown in parallel under identical conditions. Relative to the common ancestor, expression of 6.8% (∼279 genes) of the measurable transcriptome was at least 2-fold increased or decreased in the evolved isolates (Figure S2). This number favorably compares with an early proteomic analysis of these strains grown in chemostat monoculture wherein Kurlandzka et al. [31] found that ∼160 expressed proteins of the ∼700 they were able to resolve differed between evolved clones and their common ancestor.

**Figure 2:**
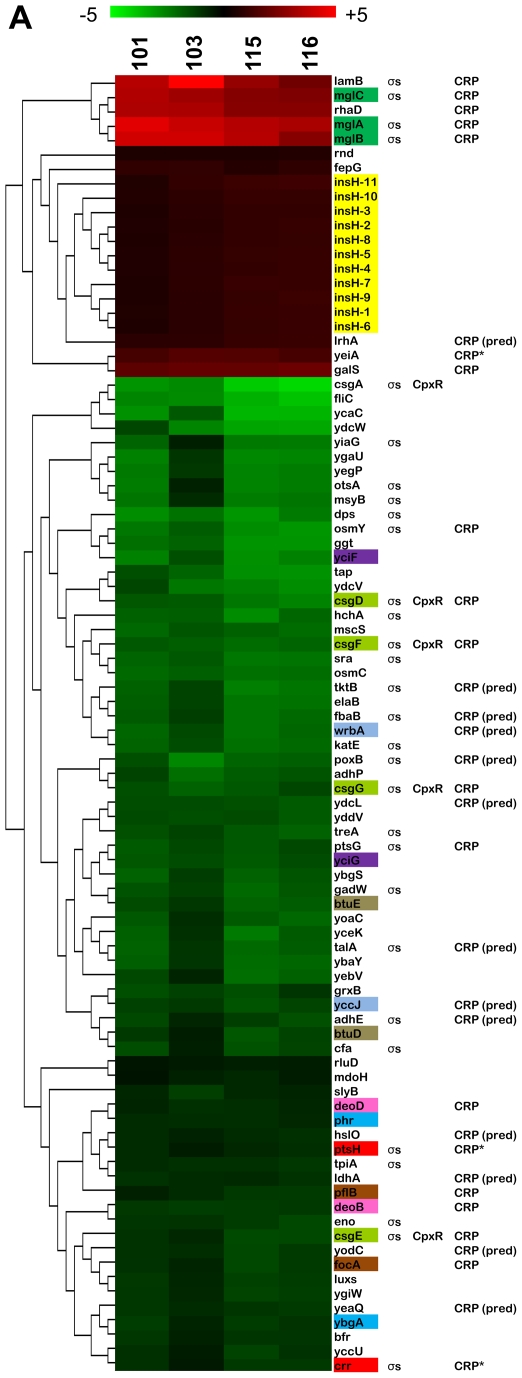
1-class SAM analysis for terminal isolates grown in chemostat monoculture. Hierarchical clustering of the 1-class SAM analysis results shows that a number of significantly up- or down-regulated genes is similarly expressed among all of the evolved isolates. The majority of these are part of the RpoS regulon. In general, genes involved in glucose transport (such as *lamB* and *mglABC*) are up-regulated while several genes involved in central metabolism are down. Biological and technical replicates are displayed as averages (means). Co-transcribed genes are color-coded. Columns to the right of each gene reflect known or predicted regulation by the two dominant global regulators, σ^S^ and CRP. Regulation by CpxR is also included to facilitate comparison with Figure 3. An asterisk (*) indicates the listed regulator is the sole known regulator for that gene. Predicted regulators are followed by the abbreviation “pred.” Regulatory information was compiled from the EcoCyc and TractorDB databases (see Materials and Methods for details). SAM analyses were performed using the TIGR MeV 4.1.01 SAM module on the full, un-averaged data set. All strains were assigned to the same class and the data were tested against a mean log_2_ ratio of 0 using the 1-class SAM design.

Using 1-class SAM, we identified 90 genes whose expression was significantly up- or down-regulated in all clones when grown in chemostat monoculture (Figure 2, Table S1). The 21 up-regulated genes, representing 9 unique transcription units, are primarily involved in carbon catabolism. The remaining 69 down-regulated genes from 58 transcription units belonged to a variety of MultiFun classes including carbon metabolism, building block/macromolecule biosynthesis, transport and adaptation to osmotic stress.

#### Genes up-regulated in all evolved strains

Four transcription units up-regulated in all evolved isolates (*lamB*, *mglBAC*, *mglD* and *rhaBAD*) are involved in carbon metabolism and are positively regulated by CRP, a major global regulator of catabolite-sensitive operons (Figure 2, Table 2) [30],[40]. *LamB* and the *mgl* operon are also regulated by the stationary phase sigma factor RpoS, and, along with *mglD* (also known as *galS*), have previously been shown to be targets of selection during long-term adaptation to glucose limitation [41],[42]. Interestingly, expression of the IS5 insertion element transposase, *insH* is also elevated in all isolates. Although the overexpression of *acs* in CV101 described by Treves et al. [39] is the result of IS30 movement, our observation of elevated *insH* is intriguing, especially considering the extent to which insertion element movement has influenced adaptation in other experimental evolution studies (as reviewed in [43]).

**Table 2:** Expression levels of selected genes from 1-class and 4-class SAM analyses.

ID	gene	SAM class	mean log_2_ CV101/JA122	mean log_2_ CV103/JA122	mean log_2_ CV115/JA122	mean log_2_ CV116/JA122	gene product	Transcription Unit	MultiFun Category
b4069	***acs***	4-class	3.9	0.0	−1.5	−0.3	acetyl-CoA synthetase	acs-yjcHG	Metabolism; Building Block Biosynthesis; Acetate utilization; Central intermediary metabolism;
b4484	***cpxP***	4-class	0.0	1.7	−0.8	0.0	reg. of Cpx response	cpxP	Cell processes; Adaptations; Regulation; 2-component regulatory system
b2417	***crr***	1-class	−0.9	−0.5	−1.1	−1.1	glucose-specific enzyme IIA component of PTS	ptsHI-crr (ptsHp1)	Metabolism; carbon utilization; The PTS Fructose-Mannitol (Fru) Family, transport; substrate; D-glucose/trehalose
b1073	***flgB***	4-class	1.3	−1.1	2.0	0.7	flagellar component of basal-body rod	flgBCDEFGHIJ	Metabolism; Macromolecule Biosynthesis; Flagellum; Motility (incl. chemotaxis, energytaxis, aerotaxis, redoxtaxis), cell structure;
b1923	***fliC***	1-class	−2.6	−2.7	−3.5	−3.8	flagellar filament structural protein (flagellin)	fliC	Metabolism; Macromolecule Biosynthesis flagella
b2151	***galS***	1-class	1.9	2.0	2.0	2.2	DNA-binding transcriptional repressor	galS	Metabolism; Carbon utilization; Regulation; Transcriptional repressor
b1732	***katE***	1-class	−1.9	−1.5	−2.2	−2.1	hydroperoxidase HPII(III) (catalase)	katE	Cell processes; Protection; Detoxification (xenobiotic metabolism)
b4036	***lamB***	1-class	3.6	5.1	2.9	2.2	maltose outer membrane porin	malK-lamB-malM (malKp)	Transport; (The Outer Membrane Porin (OMP) Functional Superfamily); The Sugar Porin (SP) Family
b3454	***livF***	4-class	0.1	−0.1	0.6	−1.5	leucine/isoleucine/valine transporter subunit	livKHMGF	Primary Active Transporters; (isoleucine/valine/leucine); amino acid transport/metabolism); ATP-binding Cassette (ABC) Superfamily
b2149	***mglA***	1-class	4.5	3.9	3.6	3.3	methyl-galactoside transporter	mglBAC (mglBp)	Metabolism; Carbon utilization; The ATP-binding Cassette (ABC) Superfamily
b0929	***ompF***	4-class	1.2	−1.7	1.3	0.0	outer membrane porin 1a (Ia;b;F)	ompF	Transport; β-barrel porins (Outer Membrane Porin (OMP) Functional Superfamily)
b1101	***ptsG***	1-class	−1.8	−1.4	−1.7	−1.4	PTS system glucose-specific IICB component	ptsG	Metabolism; Carbon utilization; Regulation; Posttranscriptional; Transport
b3461	***rpoH***	4-class	−0.2	0.8	−0.5	−0.7	RNA polymerase, σ32 (σH) factor	rpoH	Information transfer; Transcriptional Regulation; σ factors, anti–factors; adaptation to stress; temperature extremes

#### Genes down-regulated in all evolved strains

Multiple genes scored as down-regulated by 1-class SAM analysis (12) are involved in central metabolism (see Figure 2 and Figure S3). Expression of two components of the glucose-specific PTS permease, *ptsG* (EIIB/C^Glc^) and *crr*, as well as the non-specific PTS component *ptsH* (HPr) are all significantly lower even though clones leave little residual glucose and all exhibit marked improvement in glucose uptake (Table 1) [15],[44].

Five genes in the glycolytic and the pentose phosphate pathways also exhibit decreased transcript levels in the evolved isolates. These include two enzymes responsible for converting fructose-6-phosphate into glyceraldehyde-3-phosphate (*tpiA* and *fbaB*) and enolase (*eno*), which catalyzes the final step in the conversion of 2-phosphoglycerate to phosphoenolpyruvate. Interestingly, enolase plays a secondary role as part of the *E. coli* degradosome that rapidly breaks down *ptsG* mRNA in response to high levels of glucose-6-phosphate and fructose-6-phosphate [45]. Transketolase B and transaldolase A (*tktB* and *talA*), which act in the non-oxidative branch of the pentose phosphate pathway, also show decreased expression; however, both are variants of a more active isoenzyme. Thus, their down-regulation may not adversely affect pentose phosphate pathway function.

We also observed diminished expression of 7 genes that play a role in mixed acid fermentation: pyruvate oxidase (*poxB*), pyruvate formate-lyase (*pflB*), acetaldehyde dehydrogenase (*adhE*), both ethanol and alcohol dehydrogenase (*adhP* and *adhE*) and D-lactate dehydrogenase (*ldhA*). While lower transcript levels of these genes do not necessarily mean that their corresponding enzyme levels are insufficient to convert pyruvate into fermentation products under glucose limitation, the pattern of down-regulation suggests that conversion of pyruvate into acetyl-CoA most likely occurs via the pyruvate dehydrogenase complex, and that the primary fermentation product is acetate.

Finally, transcripts needed to manufacture motility and attachment structures, in particular the flagellin (*fliC*) and curlin (*csgA*) genes, also show decreased expression, an observation that is perhaps not surprising considering that the chemostat environment is well-mixed, and that attachment and motility may be of limited utility therein (Table 2).

#### Regulation of genes similarly expressed in all evolved isolates

Because it is likely that fewer than 10 periodic selection events occurred prior to establishment of stable polymorphism [14], it is reasonable to consider the possibility that many of the observed changes in gene expression involve coordinate regulation. Indeed, we found that many changes are attributable to two global regulators, σ^S^ and CRP. Strikingly, a third of the 90 up- and down-regulated genes are part of the RpoS-mediated stress response (Figure 2). This is particularly noteworthy given that loss-of-function mutations in *rpoS* are frequently encountered in both wild and experimental *E. coli* populations following prolonged exposure to low nutrient conditions [46]–[48]. Nineteen genes (21%) are regulated by CRP and an additional 13 (14%) have predicted CRP binding sites (Figure 2).

### When cultured separately, gene expression in the dominant clone, CV103, differs from the other adaptive clones

**Figure 3:**
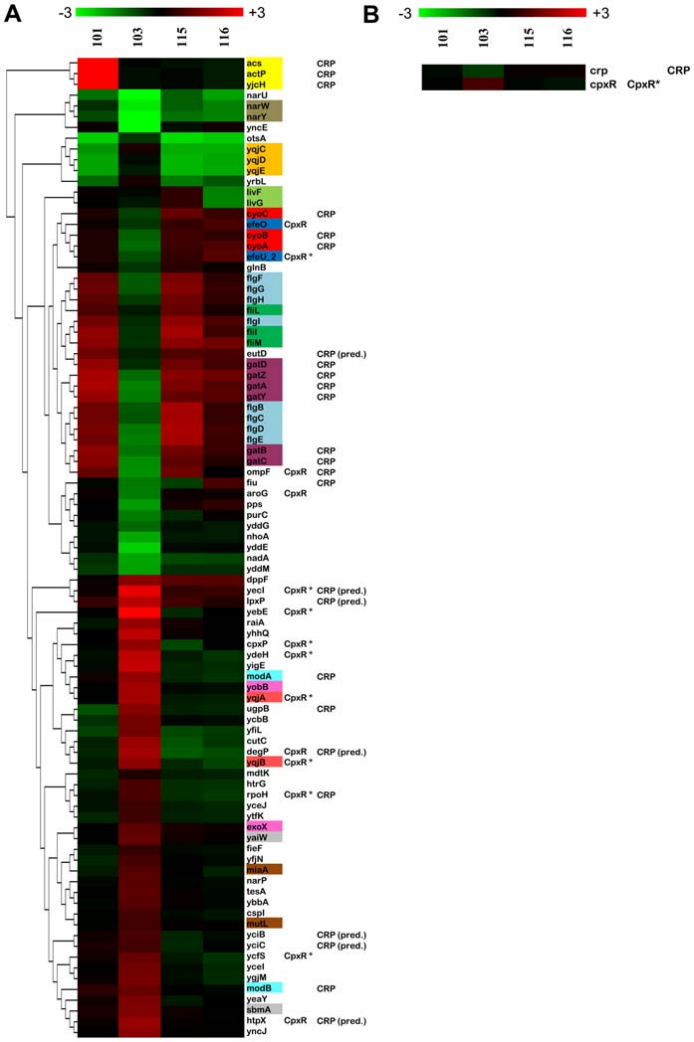
Top 91 significant genes by 4-class SAM for evolved isolates grown in chemostat monoculture. 4-class SAM analysis shows the expression profiles of CV101, CV115 and CV116 are very similar with the exception of over-expression of the acetyl CoA synthtase operon in CV101. Conversely, CV103 differs from the other evolved isolates at a number of loci. Fourteen genes from 12 transcription units are part of the CpxR regulon while 18 (8 T.U.s) are regulated by CRP. If predicted CRP binding sites are considered, then the number of genes that may respond to CRP increases to 25 (7 additional transcription units). (A) Hierarchical clustering of all 91 significant genes with biological and technical replicates displayed as averages (means) showing the difference in expression profiles between CV103 and the other three strains. (B) Average expression profile for *crp* and *cpxR*. For both (A) and (B), co-transcribed genes are color-coded. Columns to the right of each gene reflect known or predicted regulation by the two dominant global regulators for this data set, CpxR and CRP. An asterisk (*) indicates the listed regulator is the sole known regulator for that gene. Predicted regulators are followed by the abbreviation “pred.” Regulatory information was compiled from the EcoCyc and TractorDB databases (see Materials and Methods for details). SAM analyses were performed using TIGR MeV 4.1.01 SAM module on the full, unaveraged data set. For (A), the significance threshold was assigned visually after examining the plot of observed vs. expected d-values and adjusting the tuning parameter (δ) by hand to reflect a natural break in the data from a line with slope =  1. This threshold corresponded to a δ value of 0.272 and a median false-discovery rate of 0%. For (B), the significance threshold was assigned using the highest δ value that gave a median false discovery rate of 0%, an analysis that returned a total of 303 significant genes, only two of which are displayed.

To ascertain how the transcriptional profiles of evolved clones differ from one another we performed a 4-class SAM analysis (Figure 3, Table 2, Table S2). Aside from the anticipated overexpression of *acs-yjcHG* in CV101, the transcription patterns of CV101, CV115 and CV116 appear remarkably similar. By contrast, CV103 differs from the other three at a number of loci, and accounts for the great majority (∼93%) of the significant differences that distinguish adaptive clones. When we adjusted δ (a tuning parameter that can be manually adjusted) to reflect a natural break in the data, we found that a total of 91 genes from 64 transcription units significantly differ in steady state expression levels in at least one isolate at a false discovery rate of 0%. These genes tend to fall into three MultiFun classes: metabolism, cell structure and transport. Under the category of metabolism, forty-four genes from twenty-seven transcription units vary in their relative expression patterns. The metabolism-building block biosynthesis subclass contained the most independent transcription units (8/27), including *acs-yjcHG* (acetyl CoA synthetase). Conspicuously absent is mRNA transcribed from the NRZ operon (narZYWV), which is deleted in CV103. This operon is normally induced during stationary phase and appears to be actively transcribed in the ancestor but slightly down-regulated in CV101, CV115 and CV116 (Figure 3, [49]. However, the fitness effect of this deletion is currently unknown (Figure 1).

#### Genes down-regulated in CV103 that are up-regulated in CV101, CV115, and CV116

Relative to its ancestor JA122 grown in monoculture, 25 genes show diminished expression in CV103, but increased expression in the other evolved clones. Especially noteworthy note are the flagellar motor complex and flagellar hook gene transcripts, which are conspicuously down-regulated in CV103, but up-regulated in CV101, CV115 and CV116. Up-regulation of flagellar genes has been previously observed both under glucose limitation and during growth on secondary carbon sources such as acetate [50]–[53]. Thus, in certain respects CV103 exhibits a transcriptional response inconsistent with adaptation to nutrient-poor conditions. While the flagellar master switch, FlhDC, can be induced by CRP, it is also subject to repression by multiple proteins (e.g., phosphorylated OmpR) any one of which could down-regulate flagellar transcripts [54]. Interestingly, *fliC*, the gene that encodes flagellin, the flagellar structural subunit, is down-regulated in *all* isolates in the 1-class SAM analysis suggesting that, despite differences in motor complex and hook gene transcript levels, all four evolved strains are unable to make an intact flagellum. These results are supported by the observations that the other Class III flagellar genes, *che*, *mot* and *tsr*, are downregulated at the 0% FDR level (but not by the more stringent method of manually adjusting δ), and that only the ancestor displays movement when grown in motility agar (data not shown).

Multiple CRP-induced transport-related gene transcripts also showed diminished relative abundance in CV103. Both the galactitol-PTS-permease operon (part of the tagatose-6-phosphate pathway), as well as the gene for the OmpF outer membrane porin are repressed in CV103 (Table 2). The latter observation is consistent with the finding that OmpF protein expression is greatly diminished in CV103 relative to other members of the consortium and their common ancestor [31]. OmpF expression has been studied extensively in relation to culture under glucose-limitation [55]–[58]. Typically, aerobic glucose limitation leads to increased *ompF* expression as part of a general strategy by the cell to increase membrane permeability. The deviation of CV103 from this pattern is another example of how the transcriptional profile of the dominant clone grown in monoculture is unique in the context of previously characterized adaptations to nutrient limitation. While the regulation of OmpF expression is complex and involves multiple factors, it is important to note that high intracellular acetyl phosphate levels (as may exist in an acetate-secreting strain such as CV103) could down-regulate *ompF* by phosphorylating OmpR, a negative regulator of *ompF* transcription [58],[59].

#### Genes up-regulated in CV103 that are unchanged or down-regulated in CV101, CV115, and CV116

Forty genes representing 35 transcription units were significantly up-regulated in CV103 but were unchanged or down-regulated in the other evolved strains (Figure 3A). Several genes in this group function to mitigate cellular stress, perhaps most notably the heat-shock sigma factor RpoH, which is normally transcribed during carbon starvation and exposure to hyperosmotic conditions and acts as a sigma factor for five other transcription units we see up-regulated in CV103 (Table 2) [40],[60],[61]. Although not part of the RpoH regulon, two other functionally-related, CpxR-dependent genes also show increased transcript abundance in CV103: CpxP, an extra-cytoplasmic stress response regulator and potential chaperone, and DegP, a high-temperature protease/chaperone (Table 2). DegP assists in proper folding of the maltose operon regulator MalS at low temperatures (between 28°C and 37°C), which may be significant given the increased expression of the *mal* genes in all of the evolved isolates and the low temperature at which our chemostats were run (30°C) [62].

#### Expression of a small number of genes distinguishes CV101, CV115, and CV116 from one another

As noted above, the most obvious difference between CV101 and the other evolved strains is overexpression of *acs* (acetyl CoA synthetase) and *actP* (acetate/glycolate permease) (Figure 3 and Table 2). Aside from these, only a few genes distinguish CV101 from CV115 and CV116. For example, CV115 has increased expression of *livF* and *livG*, part of the leucine ABC transporter and branched-chain amino acids transporter, while CV116 displays a higher transcript level for *fieF*, a putative siderophore outer membrane receptor (Table 2).

#### Regulation of genes which are differentially expressed among evolved isolates

Genes differentially expressed among the adaptive clones are predominantly regulated by one of two global regulators. CRP regulates 23% of the 4-class SAM transcription units, while the extracytoplasmic stress response regulator CpxR regulates 19% of these transcription units (Figure 3) [63],[64].

Our global gene expression analyses are in overall agreement with previously published proteomic, biochemical and genetic data for these same isolates [14],[15],[31],[39]. *acs* overexpression by CV101 has now been confirmed by multiple lines of investigation. Also, both mRNA and protein profiling indicate that, relative to the common ancestor, up-regulation of *lamB* occurs at steady state under glucose limitation in all evolved isolates. Likewise, down-regulation of *ompF* in CV103 and its concomitant up-regulation in the other three strains is confirmed by both techniques. Lastly, although increased expression of *rpoH* (Figure 3) was not observed on 2-D gels, Kurlandzka et al. [31] did observe increased expression of σ^32^-dependent proteins such as GroES and GroEL.

### Transcriptional profiling of the evolved consortium

Reconstruction experiments demonstrated that three of the evolved strains could stably coexist in continuous culture as a consortium, and that their coexistence was made stable by cross-feeding [15]. When limited on 0.0125% glucose, the consortium reproducibly apportioned as ∼70% CV103, 20% CV116 and 10% CV101 at steady state. To better understand mechanisms underlying stable coexistence we interrogated the consortium transcriptome.

**Figure 4:**
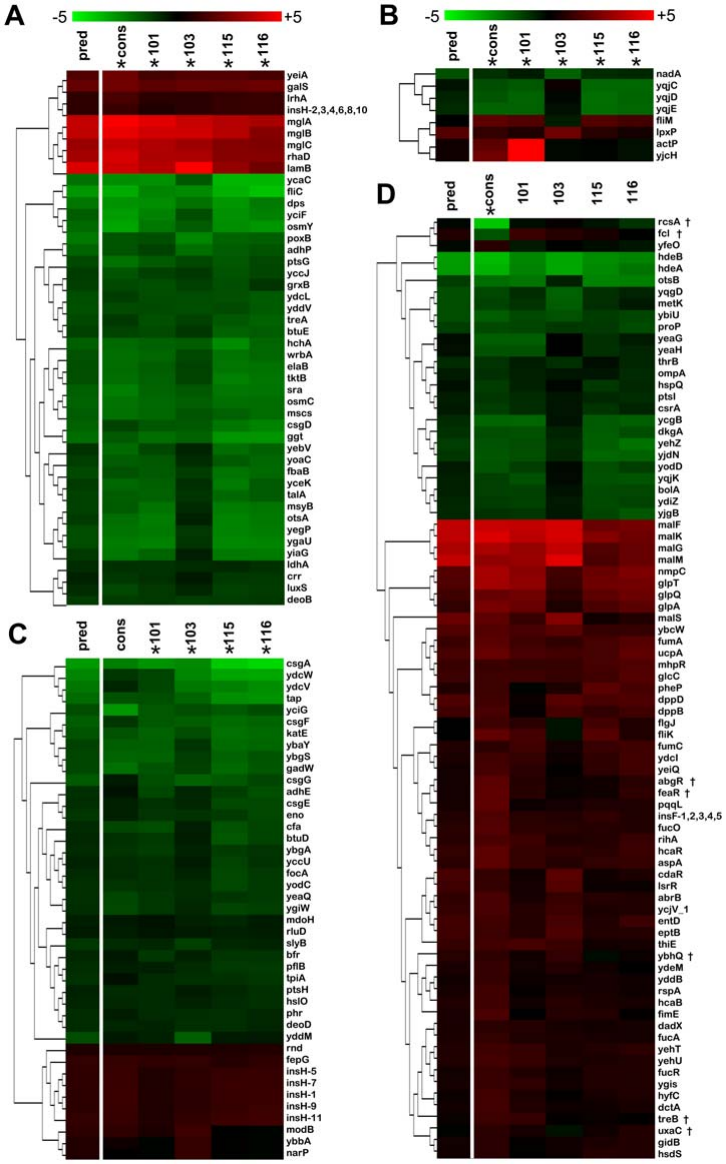
Expression profile SAM analysis of strains in co-culture reflects many, but not all, regulatory changes observed when strains are grown in monoculture. Genes found to be altered in expression by visually adjusting δ in the consortium 1-class SAM agree with predictions on monoculture results. Predicted expression levels (shown in the far left column for each heat-map) were calculated as a weighted average of monoculture log_2_ ratios under the assumption that the contribution of each strain to the total RNA pool is proportional to their relative frequency in the chemostat (i.e. 10% CV101, 20% CV116 and 70% CV103). For each panel, columns with an asterisk (*) are significant using the highly stringent method of adjusting δ (*see* Materials and Methods for details). (A) Heat-map of genes significant in both the consortium (column labeled “cons”) and monoculture 1-class SAM analyses. (B) Genes whose expression is significant in the consortium 1-class SAM analysis and the monoculture 4-class SAM analysis. (C) Genes whose expression is significant in the monoculture 4-class SAM analysis but not in the consortium 1-class SAM analysis. (D) Genes that are significant in the consortium 1-class SAM analysis but not in either of the monoculture analyses. However, the majority of genes in panel D *are* significant at the less stringent 0% false discovery rate threshold. † to the right of the gene name indicates the gene is not significant at either threshold in any of the monoculture analyses.

In general, we observe that genes significantly up or down in the 1-class SAM monoculture analysis behave similarly when clones are co-cultured (Figure 4). Furthermore, consortium profiling extends the results of our monoculture analyses to include other members of operons previously identified by 1-class SAM. For example, *malK* and *malM* (which are co-transcribed with *lamB*), as well as *malF*, *G* and *S* from two separate, but similarly regulated transcription units each show increased expression when cells are cultured as a consortium (Figure 4D).

Several transcripts significantly up-regulated in the consortium, including genes for a second glycerol-3-phosphate transporter/phosphodiesterase, *glpTQ*, part of the G3P-dehydrogenase, *glpA*, and fumarase genes, *fumA* and *fumC*, were not scored as significantly up-regulated in the monoculture 1-class SAM using the highly stringent method of hand-tuning δ (Figure 4D, Figure S3). However, the majority of these *were* considered significant when a strict 0% FDR cutoff was applied. Those that do not meet this criterion are marked with a “†” in Figure 4.

**Figure 5:**
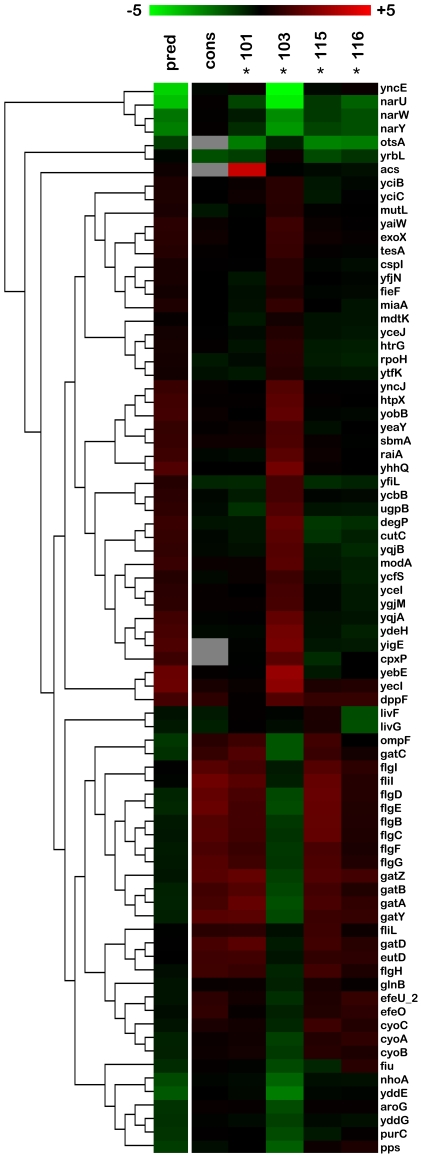
Some genes differ markedly between the monoculture and consortium expression profiles. The majority of these genes are those from the 4-class SAM analysis that distinguish CV103 from the other evolved isolates. Predicted expression levels were calculated as for Figure 4 and are shown in the far left column marked “pred.” Comparison of the consortium and predicted transcriptional profiles suggests that expression of a number of genes in CV103 changes depending on whether it is grown alone or in the presence of CV101 and CV116. Grey boxes indicate the gene was excluded from the analysis due to a lack of high-quality signal on the array.

When we compared the consortium's transcriptional profile to the 4-class SAM (Figure 5) we were surprised to find that many of the transcripts that distinguished CV103 from the other evolved clones in monoculture had expression patterns similar to CV101, CV115 and CV116, even though reconstruction experiments show that CV103 always emerges as the numerically dominant consortium member [14],[15]. To ascertain whether this phenomenon was a general feature of the dataset, we looked at transcript levels across all samples for genes that were either (A) significant in the consortium analysis but not in the monoculture experiments, or (B) significant in the monoculture experiments but not in the consortium profile. For this comparison, we used the highly stringent method of hand tuning δ to determine significance cutoff. In both cases, the vast majority of genes that were differentially regulated in CV103 monoculture (and thus distinguished this isolate from the other clones) again had transcript levels that closely matched CV101, CV115 and CV116. While this analysis is limited by the fact that the individual contributions of isolates cannot be dissected from the consortium RNA pool, the sheer number of transcripts that follow this trend strongly suggests that CV103 has a different gene expression profile in the shared metabolic environment of the consortium than when it is grown in isolation.

### Confirming expression changes for select genes by qRT–PCR

Three genes (*lamB*, *acs* and *flgB*) with different relative expression levels were selected for quantitative reverse transcriptase PCR on RNA isolated from chemostat monocultures. PCR results for all three closely approximated array results with correlation coefficients ranging from 0.78–0.99 (see Figure S4).

### Sequence analysis of likely targets of selection

To place our results in the context of previously published work and to uncover mutations which may contribute to the transcriptional profiles of the adaptive clones, we sequenced 13 candidate genes and their corresponding regulatory elements (Table 3, for primers see Table S3). Selection of candidate genes was guided by the observation that members of the evolved polymorphism had differentiated from one other and their common ancestor with respect to glucose, acetate and glycerol metabolism [15].

**Table 3:** Sequenced genes.

Locus	Gene product	MG1655 position (gene length)	transcriptional start (relative to translational start)	sequenced region relative to translational start site	mutations
*acs*	acetyl-CoA synthetase (AMP-forming)	4,283,436 ← 4,285,394 (1959 bp)	−224	CV103: −439→end +14 ; JA122, CV101, CV115 and CV116: −439→+391, +441→end+14	A→T, position −93. Shared by JA122, CV101, CV103, CV115 and CV116. CV101 also has an IS 30 element insertion in the promoter as previously reported.
*crp*	CRP transcriptional dual regulator	3,484,142→3,484,774 (633 bp)	−167	−163→547	none
*cya*	adenylate cyclase	3,989,176→3,991,722 (2547 bp)	−379	−428→end +56	none. CV115 not sequenced.
*glpK*	glycerol kinase	4,113,737 ← 4,115,245 (1509 bp)	gene internal to mRNA start	+18→end +9	Gly→Gly at aa 225 in CV116. JA122, CV101, CV103 and CV115 unchanged.
*glpR*	sn-Glycerol-3-phosphate repressor	3,557,870 ← 3,558,628 (759 bp)	−286	−25→end +23	Gly→Ala, aa 55 in JA122, CV101, CV103, CV115 and CV116.
*lamB*	maltose high-affinity receptor	4,245,994→4,247,334 (1341 bp)	gene internal to mRNA start	−16→end +241	none
*malT*	maltose operon transcriptional regulator	3,551,107→3,553,812 (2706 bp)	−61	−541→+1125	Ala→Glu, aa 53 in CV101, CV115 and CV116. JA122 and CV103 unchanged.
*mglD*	GalS transcriptional dual regulator	2,238,650 ← 2,239,690 (1041 bp)	−42	−158→end +503	G→T transversion located 3 base-pairs from the end of mglD. Shared by CV101, CV103, CV115 and CV116. Absent in JA122
*mlc*	DgsA transcriptional repressor	1,665,368 ←1,666,588 (1221 bp)	−39	−75→end +41	none
*pta*	phosphate acetyltransferase	2,412,769→2,414,913 (2145 bp)	gene internal to mRNA start	JA122: +17→+1865 ; CV101, CV103, CV116: +17→end +50	none. CV115 not sequenced.
*ptsG*	enzyme II glc	1,157,092→1,158,525 (1434 bp)	−243	−297→end +37	none
*rpoS*	RNA polymerase, sigma S (sigma 38) factor	2,864,581 ← 2,865,573 (993 bp)	−567	−185→end +48	Gln→stop aa 33 in CV101, CV103, CV115 and CV116. Unchanged in JA122.
*spoT*	GDP diphosphokinase/guanosine-3′,5′-bis(diphosphate) 3′-diphosphatase	3,820,423→3,822,531 (2109 bp)	unknown	−48→2105	none

#### Glucose transport and assimilation

Mutations that enhance the ability of *E. coli* to move glucose across the inner and outer membranes are commonly observed during adaptation to glucose limitation. Glucose can cross the outer membrane by passing through either the general porins OmpC and OmpF, or via the maltodextrin porin LamB (see Figure S3), which is part of the MalT regulon [65]. Transcriptional differences relative to the ancestor were observed for both *ompF* and *lamB* as well as a number of the other malT regulon genes (see Figure 2, Figure 4D). As OmpF regulation is complex and involves multiple regulators (any of which might be a mutational target), sequencing efforts were focused on the LamB structural gene, the *mal* transcriptional activator MalT and the *mal* repressor Mlc.

Other groups have reported that adaptive mutations in the first ∼360 amino acids of MalT eliminate the need for maltotriose inducer and thus allow continuous induction of the *mal* genes [41],[66],[67]. Likewise, mutations in Mlc that abolish repressor activity and lead to increased transcription by MalT are also common under glucose limitation [41]. Despite the fact that up-regulation of the *malEFG*, *malK-lamB-malM* and *malS* transcription units in the monoculture and consortium SAM analyses strongly pointed to increased transcription of the entire *mal* regulon in all of the evolved isolates, we were surprised to find that there were no mutations in *mlc* for any of the isolates or in the promoter region/structural gene for *malT* in CV103 or JA122. Similarly, the LamB gene itself was also unchanged across all isolates. However, when we sequenced the same portion of *malT* for the remaining strains, we identified an A→E substitution at amino acid 53 in CV101, CV115 and CV116. This region of the protein (from amino acid 44–55) forms a helix that is positioned between two ATP binding motifs and is part of a larger and widely-recognized nucleotide-binding P-loop NTPase domain [68]. Whereas most members of this family of NTPases typically have a non-polar residue at this position, CV101, CV115 and CV116 have acquired a polar substitution. The mechanistic significance of this substitution is currently unknown.

From the *E. coli* periplasm, glucose can cross the inner membrane via the phosphotransferase system (PTS), the glucose/galactose transporter MglBAC and/or the galactose MFS transporter GalP (Figure S3). In our 1-class SAM analyses, the PTS enzyme II^glc^ (PtsG/Crr) and the MglBAC transporter were differentially transcribed, with II^glc^ being repressed and MglBAC upregulated. While mutations in *ptsG* do confer a moderate fitness advantage in glucose-limited chemostat culture, up-regulation of MglBAC, either by inactivating its repressor, MglD, or eliminating the repressor binding site in the mgl operator, exerts a much greater effect on glucose transport [42],[69],[70]. As no mutations in GalP under glucose limitation have been reported in the literature, we focused our sequencing efforts on the *ptsG* structural gene and its upstream regulatory region, the *mgl* transcriptional repressor *mglD*, and the *mgl* operator sequence *mglO*. As might be expected from their relative activities, no mutations were found in the *ptsG* structural gene or its upstream regulatory sequence for any of the evolved isolates, but they all shared the same mutation in the *mgl* operator: a single G→T transversion located 3 base-pairs from the end of *mglD* (Table 3). This substitution is identical to one previously reported [42] and lies within the repressor binding site, thus allowing semi-constitutive transcription of *mglBAC* and increased transport of glucose into the cytoplasm.

#### Acetate uptake and secretion

The basis of the acetate-scavenging behavior of CV101was previously identified to be IS element-mediated, constitutive over-expression of acetyl-CoA synthetase [39]. Re-sequencing of the acs gene and its promoter region confirmed the presence of the IS element insertion in CV101, but also highlighted the importance of the ancestral promoter composition relative to the fully sequenced E. coli K-12 strain MG1655: JA122, CV103, CV115 and CV116 all share an A→T substitution at position −93 relative to the *acs* start site (Table 3). While the ancestral sequence of the *acs* promoter region was accurately determined by Treves et al. [39], at the time of publication JA122 was considered the “wild-type” condition when in fact the opposite is true: the A at position −93 is conserved across the *E. coli* clade of the *Enterobacteriaceae*. This phylogenetically-related group contains genera (*Citrobacter*, *Shigella*, *Salmonella* and *Escherichia*) that live almost exclusively in the gastrointestinal tract of warm-blooded mammals, an environment in which extracellular acetate is an important source of carbon [71]. The base pair in question lies in the first of two CRP binding sites for the proximal *acs* promoter P2. This CRP binding site is required for full induction of acetyl CoA synthetase; directed point mutations in this region do not eliminate *acs* expression but can cause a 40–80% decrease in transcription [72]. In the absence of a constitutive mutation such as the IS-element insertion in CV101, transcriptional control of this locus is thought to occur primarily via induction. This induction is sensitive to the level of cAMP in the cell, i.e. higher cAMP concentrations (in conjunction with CRP) appear to stimulate *acs* expression [73]. The average level of CRP transcript compared to the ancestor is slightly lower in CV103 versus the other isolates at the 0% FDR threshold cutoff (Figure 3B). Taken together, these data strongly suggest that the ancestor, as well as CV103, CV115 and CV116 exhibit *less* than wild-type expression of *acs* and that the induction of this operon in CV103 may be inhibited by higher glucose consumption and/or lower levels of CRP. Restoring base pair −93 to the wild-type state is all that is required to generate an acetate scavenging strain that can stably co-exist with CV103 [39]. No additional changes were found in either the promoter region or the *acs* gene for any of the isolates, with the caveat that a ∼50 base pair segment of the *acs* sequence of JA122, CV115 and CV116 (between nucleotides 391 and 441) was not available due to a technical failure. However, it is unlikely that this region contains a mutation, as the sequence for CV103 is identical to that of the reference strain MG1655.

*E. coli* can excrete excess acetate via the phosphotransacetylase/acetate kinase (*pta*/*ackA*) pathway when carbon catabolism generates more acetyl CoA than can be efficiently utilized by the TCA cycle or other acetyl-CoA consuming pathways [71]. Given that CV103 scavenges more glucose and accumulates more acetate in batch and chemostat monoculture than either its ancestor or CV116, and given that the kinetics of acetate kinase are comparable between all of the isolates in chemostat culture [15], we sequenced the gene for phosphotransacetylase (*pta*). However, no mutations were found among any of the strains with the caveat that we were not able to capture the first 17 bp of the gene.

#### Glycerol and Glycerol-3-phosphate metabolism

Rosenzweig et al. [15] presented enzyme kinetic data suggesting differential metabolism of glycerol by CV116 relative to CV101 and CV103. Glycerol amendment of the media used to feed the evolved consortium altered clone frequencies as predicted by those data. We speculated that a mutation in glycerol kinase (*glpK*) could explain these observations. Sequencing of *glpK* did uncover a single point-mutation in CV116; however, as this was a silent substitution (glycine→glycine at amino acid 225), we cannot argue that the mutation has adaptive significance (Table 3). Mutations in the glycerol-3-phosphate regulon repressor, GlpR, could also account for enhanced glycerol metabolism by CV116. Sequencing of this gene revealed a glycine→alanine substitution at amino acid 55 in *all* of the isolates, including the ancestor. After re-examining the ancestry of JA122, we found that the glpR mutation could be traced back to its progenitor *E. coli* K12 strain, C600. While this is a fairly modest mutation, it occurs at a highly conserved position and has been previously reported to result in constitutive expression of genes involved in glycerol utilization [74]–[76]. Despite this mutation, the regulon is still subject to glucose-mediated catabolite repression at the transcriptional level, as well as post-translational inhibition by IIA^glc^ (*crr*, downregulated in all evolved strains) and fructose-1,6-bisphosphate [75],[77]. In regard to the behavior of our isolates, the activity of the glycerol kinase enzyme (*glpK*) and glycerol-3-phosphate dehydrogenase (*glpD*) are lower in CV103 relative to its ancestor and the other evolved strains [15]. However, no significant differences in *glpK*, *F* (the glycerol facilitator), or *D* expression were detected between the parent and evolved strains on our arrays, as would be expected if the operon is constitutively active across all isolates.

Conversely, *glpT* (the glycerol-3-phosphate transporter), *glp Q*, and *glpA* (a subunit of anaerobic glycerol-3-phosphate dehydrogenase) are significantly upregulated in all of the evolved clones in the 1-class community analyses, as well as at the 0%-FDR level in monoculture. Considering that the ancestor likely has constitutive expression of *glpT*, this further increase is surprising but not inconsistent with the observation that either glycerol or glycerol-3-phosphate cross-feeding maintains the CV103/CV116 equilibrium [15].

#### Global regulators of carbon metabolism

Considering the large number of coordinately transcribed genes whose expression levels differed significantly in the evolved strains relative to their ancestor, we strongly suspected that alterations in global regulatory pathways had occurred during the course of the evolution experiment. As 39% of the down-regulated genes in the 1-class analysis are part of the σ^S^ regulon, and mutations in *rpoS* have been repeatedly observed in glucose-limited chemostat cultures, we sequenced this gene [47],[78]. We found that all evolved isolates shared a C→T transition at nucleotide 97 that resulted in an a.a.33 Q→amber mutation. Given the severe nature of the resulting truncation, it is likely that this mutation negatively affects σ^S^ activity. Interestingly, the *rpoS_Am_* mutation at this position has been observed in a number of other *E. coli* isolates [79]. In suppressor-free strains that carry the *rpoS_Am_* mutation, translation of a truncated Δ1–53 σ^S^ can proceed from a downstream secondary translation initiation region [80]. This shortened RpoS, while not fully functional, retains partial activity and appears to have a preference for supercoiled promoters [81],[82]. In light of these observations and because our experimental strains carry the supE44 amber suppressor, we performed catalase and glycogen staining assays to test whether or not our evolved isolates retained any σ^S^ activity. Compared to their common ancestor, all four of the evolved strains showed reduced catalase activity (weak bubbling after more than 5 seconds of exposure to H_2_O_2_), as well as impaired ability to accumulate glycogen (little to no staining with iodine), indicating that σ^S^ activity was indeed diminished. These observations are in concordance with our expression profiling results in general and with the reduced expression of *katE* (catalase HPII) in particular (see Figure 2).

Many genes from our expression analyses are also known to be regulated by cAMP-CRP. However, sequencing did not reveal any mutations in the promoter regions or structural genes for CRP or adenylate cyclase (the enzyme that catalyzes the formation of cAMP).

## Discussion

Genetic polymorphism pervades most populations, and various balancing mechanisms have been invoked to explain how diverse genotypes can be stably maintained over successive generations [see 83]. Some, such as differential selection on the sexes or on different life stages do not apply to bacterial populations, while others, such as differential selection in space or time not only do, but can be empirically tested in the laboratory [22],[84],[85]. The conditions of continuous nutrient limitation in a well-mixed chemostat do not restrict the supply of mutations to a microbial population. But competitive exclusion and periodic selection could make the appearance of polymorphism an artifact of discretely sampling a continuous process. Nevertheless, multiple genotypes demonstrably persist in this simplest of laboratory environments [14],[57],[86]. The mechanisms which sustain their co-existence can be said to fall into two general categories: negative density-dependent interactions such as clonal interference [e.g.86],[87],[88], and positive density-dependent interactions such as those described by Helling et al. [14]. Because basic ecological theory predicts that the latter of these is more stable [89], we propose that the system we are studying can serve as a general model for how biodiversity arises in clonal species, how many arise from one (*e unibus plurum*).

Our approach has been to combine microarray-based comparative genome hybridization, transcriptional profiling, and targeted gene sequencing to understand mechanistically how multiple genotypes arise and coexist in a simple unstructured environment [14],[15],[31],[39]. Previous studies showed that coexistence arose from cross-feeding interactions in which the limiting resource was incompletely metabolized by the dominant clone, effectively creating secondary resources for niche specialists. When co-evolved clones were grown separately they differed from their common ancestor in ca. 20% of identifiable expressed proteins [31]. This observation coupled with the apparent fixation of no more than 8 adaptive mutations [14] suggested that global regulatory mutations were at least partly responsible for adaptive phenotypes. The genetic basis for sub-dominant clone CV101's ability to scavenge acetate was shown to be a regulatory mutation altering expression of the acetyl Co-A synthetase operon [39]. Completely unknown, however, are the genetic mechanisms that could explain why all adaptive clones are better at assimilating glucose than their common ancestor, why the dominant clone, CV103, does not re-assimilate residual metabolites, and how CV103 and CV116 can stably coexist. Also unknown are whether data obtained by analyzing clones separately can explain their behavior as a consortium, and how the founder genotype might have set the evolutionary trajectory taken by this population.

### Adaptation to glucose limitation: strategies and mutations shared by all evolved clones

Our results show that all evolved clones share a common regulatory response to long-term glucose limitation. In general, genes involved in the phosphotransferase system, glycolysis, the pentose-phosphate pathway and mixed acid fermentation are down-regulated whereas TCA cycle genes are up-regulated (Figure S3). At first glance it may seem that reduced expression of glycolytic genes would be disadvantageous under glucose limitation. However, consistent with the energy conservation hypothesis [e.g. see 90], it may be economical for chemostat-grown cells to synthesize the minimum level of enzymes needed to process a limiting nutrient whose residual concentration has become vanishingly low. Strikingly similar changes in central metabolic gene expression have been reported for *E. coli* in batch culture as well as for Baker's yeast following adaptive evolution in long-term, aerobic, glucose-limited chemostat culture [18],[91],[92]. The generality of this phenomenon across replicate experiments within the same species, as well as across Domains, suggests that microbes may have limited options for increasing fitness in environments where glucose is the sole carbon source. However, new evolutionary opportunities may arise in the form of other carbon sources released during glucose metabolism.

Our 1-class microarray analysis and sequencing results indicate that changes in levels of the stationary-phase sigma factor, σ^S^, expected from the shared C→T transition at nucleotide 97, account for many genes being significantly down-regulated in all strains. Most of these changes are consistent with the expression profiles of an *rpoS* knockout batch-cultured in rich medium: there, relative to wild type, all central metabolic pathways including the TCA cycle were down-regulated during early stationary phase, while the TCA cycle was strongly up-regulated during exponential phase [44]. At steady state under continuous nutrient limitation, bacterial growth approximates late exponential/early stationary phase in batch culture [26]. It is tempting to speculate that the pattern of expression we observe for genes in central metabolism is what might observe if an *rpoS* knockout were grown under our experimental conditions. And indeed, experiments to test this hypothesis are planned. Alternatively, up-regulation of TCA cycle genes in our strains may result from altered σ^s^ activity caused by incomplete suppression of the *rpoS_Am_* mutation, translation of truncated σ^S^, or the effect of yet-to-be identified regulatory mutation(s).

In addition to shared global expression patterns for central metabolic genes, our microarray results show that evolved isolates also up-regulate genes involved in moving glucose across the outer and inner membranes. Increased transcription of the inner membrane Mgl galactose ABC-transporter (which also transports glucose) is common response to continuous glucose limitation [42],[93], and our experimental system is no exception. This regulatory adjustment is easily accounted for by a mutation present in all of the evolved isolates in the *mgl* operator sequence that presumably interferes with GalS-mediated suppression of *mgl* transcription [42],[93]. Similarly, increased movement of glucose into the periplasm in the evolved isolates is undoubtedly due in part to overexpression of the LamB glycoporin, another hallmark feature of *E. coli* adaptation to glucose limitation [41],[94].

In Ferenci and colleagues' experiments, adaptive overexpression of LamB (which is part of the malT regulon) results from mutations in the *mal* repressor Mlc and/or its activator MalT [41],[93]. Sequencing of *mlc* and its associated regulatory region failed to uncover mutations in any of our evolved clones. We did find a mutation in the gene encoding MalT, but its distribution was limited to CV101, CV115 and CV116 and its location was unique relative to other MalT mutations characterized as constitutive. It is surprising that this mutation does not occur in CV103 considering that, on average, CV103 has 3–6 fold higher transcript levels of *lamB* than the other three strains (significant in a between-subjects t-test, p = 0.0007).

**Figure 6:**
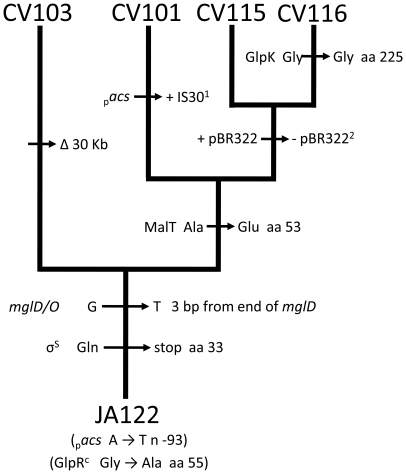
Cladogram depicting likely evolutionary relationships among CV101, CV103, CV116, and CV115. Molecular differences that distinguish clones from one another were used to reconstruct the most likely evolutionary relationships between all four of the derived isolates. Mutations identified by sequencing of targeted genes indicate that CV101, CV115, and CV116 all evolved from a CV103-like ancestor that had already acquired mutations beneficial under glucose limitation. Assuming the loss of plasmid pBR322 occurred only once during the experiment, CV115 and CV116 appear to be more closely related to one another than they are to CV101 or CV103. The branching order depicted is congruent with the branching order determined by hierarchical clustering of the expression profiles as well as phylogenies previously reconstructed from protein expression patterns which place CV103 closest to the ancestor [14],[15],[39].

While the superior glucose scavenging ability of CV103 may be attributed to its increased LamB expression relative to other evolved clones, this increase cannot be explained by inactivation of Mlc or by a constitutive mutation in MalT, as neither occurs in this strain. We also failed to recover mutations at *ptsG*, and we did not detect increased OmpF expression, both of which have been observed to enhance glucose uptake in other evolution experiments [57]. While increased LamB expression in all the evolved isolates is almost certainly due to defective *rpoS*, the *rpoS* mutation is shared and cannot account for among-strain differences [47]. CV103 does lack the Ala→Glu substitution at aa 53 in MalT (total length, 901 aa), a positive regulator of *lamB*. Based on the distribution of mutations in *rpoS*, *galS*, *acs* and *glpK*, it is highly probable that this mutation occurred in the common ancestor of CV101 and CV116 prior to specialization of CV101 on acetate, but after the divergence of CV103 (see Figure 6). Other mutations in the N-terminal portion of MalT which have been reported to arise in glucose-limited chemostats result in its constitutive expression [41]. However, despite the relatively large number of such mutations which have been characterized (at least 16), none is in the same position or motif as the one we report here [41],[68]. Interestingly, adaptation to long-term glucose limitation in batch culture can select for mutations that partially or fully inactivate MalT, one of which *does* occur in the same helix as our mutation [95]. If the *malT* mutation shared by CV101 and CV116 results in a weakened activator, and consequently less LamB, there exists the intriguing (although highly speculative) possibility that in our experiment, down-regulation of glucose influx through LamB could provide an advantage to minority clones that specialize on excess excreted carbon. Additional experiments will be needed to determine whether the *malT* mutation shared by CV101 and CV116 explains their diminished *lamB* expression, relative to CV103. Alternatively, it may be that CV103 has higher levels of endogenous maltotriose inducer, or harbors as yet unidentified mutations that affect *lamB* transcription and/or glucose uptake via other routes. Whether physiological or genetic, the mechanism underlying two-fold differences in the expression of this key transporter promises to be unique and interesting, and will be the subject of future investigations.

### The evolution of cross-feeding between CV101 and CV103

The constitutive overexpression of acetyl-CoA synthetase that enables CV101 to capture overflow acetate from the dominant clone has a clearly documented mutational basis that has been re-confirmed by our microarray and sequencing results. This mutation is selectively favored because the dominant clone, CV103, poorly recovers acetate produced via glycolysis [14], a phenotype that manifests as high equilibrium acetate concentration when CV103 is grown in chemostat monoculture (see Table 1) and absence of Acs activity when CV103 is grown in either batch or chemostat culture [15]. Given that the ancestor, JA122, has a weakened *acs* promoter, and that acetate is scavenged at low concentrations almost exclusively via the *acs* pathway, the acetate defect in CV103 could be explained by this genetic predisposition compounded by increased catabolite repression of *acs* arising from increased glucose transport. The rate of glucose uptake, equilibrium acetate concentration, and acetyl CoA synthetase measurements of CV116 under glucose limitation support this contention since all are intermediate between JA122 and CV103 (see Table 1). Moreover, when cells are grown in the presence of acetate and glycerol CV116 exhibits ancestral levels of Acs specific activity while CV103 Acs activity is negligible [15]. Thus, *acs* is neither appropriately activated nor repressed in CV103.

Regulation of *acs* expression in *E. coli* is quite complex, integrating signals from the TCA cycle, glyoxylate bypass pathway, and phosphotransacetylase/acetate kinase (*pta/ackA*) acetate dissimilation pathway [71],[73],[96]. *acs* expression can also be regulated by growth phase via Fis [97] or via the PTS system by means of cAMP-CRP [72]. Previous work indicated no defect in the regulation or structure of *ackA* [15]. In the present study, we sequenced the promoter and full structural gene for *acs* as well as the other enzyme in the dissimilation pathway, *pta*. With the exception of the first 17 base pairs of *pta* (which were not sequenced), we found no mutations. Thus, the genetic basis for loss of *acs* activity in CV103 remains obscure.

### The evolution of cross-feeding between CV103 and CV116

Increased glycerol uptake coupled with the observation that addition of glycerol increases the equilibrium frequency of CV116 co-cultured with CV103 led to the conclusion that CV116's success in the chemostat was due, at least in part, to glycerol cross-feeding [15]. Sequencing of the glycerol kinase gene (the rate limiting step in extracellular glycerol metabolism) identified a mutation in CV116 not found in the other isolates. However, given that this was a silent substitution resulting in a codon change from an abundant to a rare tRNA, and given that the surrounding sequence bears little similarity to a glycerol repressor (glpR) binding site, it is difficult to argue that that this mutation has adaptive significance. We therefore next examined glpR and were surprised to find a mutation that was not only present in the ancestor but was present in the *E. coli* progenitor strain from which JA122 was derived. This mutation has been characterized by other groups, and results in constitutive expression of the glycerol regulon [76]. Many GlpR-regulated genes did not show appreciable expression differences on our microarrays, as would be expected if they were also upregulated in the ancestor. But three genes did show modestly increased transcript levels across all evolved isolates at the 0% FDR level: the glycerol-3-phosphate transporter (*glpT*), the glycerophosphoryl diester phosphodiesterase (*glpQ*), and the anaerobic glycerol-3-phosphate dehydrogenase (*glpA*). These genes are partially under the control of GlpR, but they also have additional regulators not shared by other genes in the glycerol regulon. It appears likely that the superior ability of CV116 to recover and metabolize extracellular glycerol-3-phosphate is related to the increased expression of *glpT*, but the reason that it is able to scavenge glycerol better than CV101 and CV103 is unresolved. Catabolite repression, and/or glycolytic intermediate feedback due to increased glucose consumption may modulate GlpT activity post-transcriptionally in CV103.

### The consortium expression profile does not recapitulate monoculture profiles

Transcriptional profiling of the consortium RNA pool led to the unexpected observation that, in monoculture, CV103 has a different pattern of gene expression than when co-cultured with CV101 and CV116. The genes primarily affected are those that distinguish CV103 from the other clones in the 4-class SAM analysis, suggesting that a global regulatory mechanism is responsible for the shift in expression. Two global regulators dominate the 4-class SAM analysis, CRP and CpxR; together these explain expression patterns for nearly half the transcription units which distinguish CV103. CRP is known or predicted to influence the expression of 23% of CV103-specific transcription units, though none of these are under the exclusive control of CRP. While CpxR controls a smaller proportion of CV103-specifc transcription units, (19%), most of these are *solely* regulated by CpxR. Thus, CpxR regulation underlies much of CV103's expression pattern in monoculture; this effect is reversed when CV103 is co-cultured with the subdominant clones.

One dramatic environmental difference between the glucose-limited CV103 monoculture environment and the consortium environment is the concentration of extracellular acetate. When CV101 is present, acetate is efficiently scavenged and cannot accumulate. CpxR in its phosphorylated form mediates a global response to extracytoplasmic stressors such as high osmolarity, misfolded outer membrane protein, or alkaline pH (as reviewed in [98]). CpxR is normally activated by its sensor kinase CpxA, but it can also be phosphorylated in a CpxA-independent manner, albeit at a rate of phosphotransfer much lower than that which occurs between the sensor kinase and its response regulator. Although there have been no reports of a direct connection between extracellular acetate concentration and CpxR activation, CpxR can be phosphorylated by acetyl-P, the high-energy intermediate of the Pta/AckA pathway that accumulates during exponential phase growth on glucose and/or a proposed sensor kinase (SKx) that is connected to the Pta-AckA pathway [99]–[102]. Regardless of the precise molecular nature of the interaction, it seems clear that CpxR activation is intimately connected to acetate dissimilation. We previously reported that the K*_m_* for acetate kinase in CV103 and CV116 was lower than that of JA122 and CV101 [15]. Given the low equilibrium acetate concentration in the chemostat, it was concluded that this decrease in *K_m_* should not significantly affect acetate uptake or secretion. However, alterations in acetate kinase activity, increased acetate secretion, or reduced acetate uptake could conceivably affect the overall performance of the Pta-AckA pathway and thus influence intracellular levels of acetyl-P and/or some yet-to-be-identified effector molecule [102]. Such interactions could be reasonably postulated to elicit a CpxR-mediated transcriptional response when extracellular acetate concentrations increase (as in CV103 monoculture).

### Founder genotype constrains evolutionary trajectories

Shared mutations in *rpoS* and *mglD* strongly support the hypothesis that competition for the limiting nutrient, glucose, was the primary selective force operating in the chemostat prior to metabolic divergence of CV101 and CV116 [15]. Increased glucose consumption coupled with acetate and glycerol secretion by CV103 created a favorable environment for the evolution of clones that could efficiently consume these two overflow metabolites. While screening for mutations that contributed to the emergence of cross-feeding populations, we unexpectedly encountered ancestral regulatory mutations in both the acetate and glycerol metabolic pathways that affect the induction of acetyl CoA synthestase (the primary acetate scavenging pathway) and the glycerol regulon repressor GlpR. As a result, it appears that the ancestor is unable to efficiently recover excreted acetate and constitutively overexpresses the glycerol regulon. We believe that these two mutations in the ancestor profoundly influenced the evolutionary outcome of these experiments (as well as the replicate evolution experiments reported in [39], which showed similar qualitative results). Impaired acetate scavenging by the progenitor of CV103 undoubtedly accelerated or predisposed the evolution of a strain that could efficiently utilize this substrate. We cannot argue that acetate scavenging clones would not have eventually arisen from a purely “wild-type” inoculum, but the repeatability of their emergence as well as the precise way in which they were invariably generated (activation of *acs* by reversion of the ancestral mutation or IS element insertion) suggests that there was strong selective pressure for changes at the *acs* locus. The influence of the ancestral GlpR mutation is less clear: Overexpression of the glycerol dissimilation pathway could affect the excretion of glycerol-3-phosphate by CV103 or enhance the ability CV116 to recover it. In either case, it seems unlikely that the presence of the GlpR mutation is mere coincidence.

Overall, the influence of mutations with global and small-scale regulatory effects on the evolution of the consortium is clear (see Figure 6). The first steps in adaptation to limiting glucose occurred via mutations that increase glucose consumption: inactivation of the stationary-phase sigma factor σ^S^ and modification of the glucose/galactose transporter MglBAC repressor binding site. Mutations at these same *trans-* and *cis-*acting elements have been previously shown to confer fitness advantages under glucose-limitation, and *rpoS* mutants are commonly found in natural *E. coli* populations [46]. Subsequently, mutation of the maltose operon activator (MalT) and deletion of the chromosomal region that contains the NarZ nitrate reductase resulted in two distinct lineages: CV103 and the progenitor of CV101, CV115 and CV116. Strain CV101 acquired the ability to scavenge excreted acetate via the insertion of an IS30 element in the promoter of the acetyl CoA synthetase gene. Two other mutations of unknown effect, the loss of the plasmid pBR322 and a silent mutation in the glycerol kinase gene *glpK*, further delineated the glycerol-scavenging strain CV116.

The founder effect is generally disregarded in microbial evolution experiments because immense population sizes enable a pool of variants to be rapidly generated by mutation and also buffer against severe genetic bottlenecks. The results presented here suggest that microbial evolution experiments *are* influenced by founder genotype and that such influences can promote evolution of stable polymorphisms.

At least one mutation instrumental in the evolution and maintenance of cross-feeding (the *acs* IS30 insertion) was compensatory rather than neomorphic. Thus, the exploration of new biochemical opportunities required recovery of old functions, in addition to the development of novel traits. These observations may not be confined strictly to experimental systems as many natural microbial populations (such as those that cause nosocomial or chronic infections) are also founded by clones. For example, chronic *Pseudomonas aeruginosa* infection of the lungs of cystic fibrosis patients frequently originates from one or a few isolates that undergo clonal expansion over the course of many years [103],[104]. Common targets of selection during adaptation of these clones to the CF lung environment are regulatory: mutations in the aminoglycoside efflux pump regulator *mexZ* can enhance antibiotic resistance and mutations in *lasR*, a regulator of quorum sensing, may influence biofilm formation during infection. Similarly, *Helicobacter pylori* infections, the cause of most gastric ulcers, are often initiated in early childhood and persist throughout the lifetime of an untreated individual [105]. In both cases, mechanistic understanding of microbial adaptation is essential to successful implementation of novel therapeutic regimens.

Transcriptional profiling and targeted gene sequencing expanded and confirmed certain aspects of our understanding of the mechanisms that drive adaptation and diversification. All identified nonsynonymous mutations were regulatory in nature, but not strictly confined to global regulators. Initial selection in the chemostat favored mutations that enhance competitive acquisition of the limiting resource (such as those in *rpoS* and *mgl*), but ancestral regulatory mutations like those in *acs* and perhaps *glpR* explain much of the unique behavior of this system. The transcriptional effect of some adaptations was apparent even when consortium members were grown in isolation, while the expression of others appeared to depend on the metabolic activity of sibling clones. Finally, even under strong selection, at least one of the most beneficial mutations served to restore a lost function, thereby creating a stable cross-feeding interaction between adaptive clones.

### Conclusion/summary

The advantages of *E. coli* as a model organism for experimental evolution lie in its ease of cultivation, large population sizes, rich history of investigation, and perceived simplicity of adaptive response. An attempt to understand how one *E. coli* clone adapts to a single environmental factor led to the unexpected discovery that out of one can come many (*e unum pluribus*), and that biological diversity can evolve and endure even under the simplest conditions.

The mutations which we have so far discovered that help to explain this phenomenon localize to transcription factors or *cis*-regulatory regions, emphasizing the profound influence of differential gene regulation on adaptive evolution. Out of necessity, previous efforts to analyze this microbiological consortium relied upon the assumption that the sum of the individual units was mechanistically equal to the behavior of the whole. And indeed, detailed analysis of each member in isolation provided useful information about both their shared evolutionary history and individual adaptive strategies. However, treating the intact consortium as a single unit revealed a transcriptomic behavior that was clearly different from a simple aggregation of its “atomized” parts (*sensu* Gould and Lewontin, [106]). Future experiments which rely on advances in whole genome sequencing, cell labeling and cell sorting will enable us to dissect the consortium into its individual components prior to analysis, and precisely identify the characteristics that define each clone's adaptive strategy. The challenge of deconvoluting individual metabolic responses in this system underscores the complexity of even a simple three-membered “community.” Our finding that the sum activities of the community do not strictly equal its parts makes clear that experimental microbial evolution is a powerful tool to study the evolution of emergent properties in complex biological systems.

## Materials and Methods

### Strains, media, and culture conditions

*Escherichia coli* JA122, CV101, CV103, CV115 and CV116 were stored at −80°C in 20% glycerol (See Table 1). Davis minimal media was used for all liquid cultures with 0.025% glucose added for batch cultures and 0.0125% for chemostats [107]. Inocula for chemostat cultures were prepared by growing isolated colonies from Tryptone Agar (TA) plates in Davis medium for 16–20 hours at 30°C, pelleting the cells at 2000× g and resuspending the pellet in fresh medium. A portion of this suspension was used to inoculate chemostats to a density that approximated the expected steady-state density. Chemostats contained Davis minimal media with 0.0125% glucose and were maintained at 30°C at a dilution rate of ≈0.2/hr for 70 hours (∼14 generations). A_600_ readings and spread plate cell counts were taken at regular intervals to monitor growth and cell densities at 70 hours were between 1.5 and 2.5×10^8^ cells mL^−1^. At the end of each chemostat run, three aliquots of 40 mL of culture were rapidly filtered onto 0.2 µm nylon membranes, flash-frozen in liquid nitrogen and stored at −80°C for RNA extraction.

For transcriptional profiling, each strain was grown in triplicate on three different occasions with independently prepared batches of media. To reduce the effect of variation in media preparation, cultures of ancestral JA122 were grown concomitantly, such that each experimental chemostat had a corresponding reference control fed off of the same media reservoir.

### Nucleic acid extraction

Genomic DNA was extracted from cells grown in batch culture using a modification of methods described [108]. Subsequent to DNA precipitation, spun pellets were re-suspended in 1XTE (10 mM Tris, 1 mM EDTA, pH 8.0) containing 50µg/mL DNAse-free RNAse A and incubated at 37°C for 30 minutes. Samples were re-extracted once with phenol∶chloroform (3∶1), once with phenol∶chloroform (1∶1) and twice with chloroform and then precipitated with EtOH using standard techniques. Following re-precipitation, the DNA was dissolved in TE.

Total RNA was extracted using an SDS lysis/hot phenol method developed by the Dunham lab http://www.genomics.princeton.edu/dunham/MDyeastRNA.htm. Briefly, frozen filters were mixed with 4 mL lysis solution (10 mM EDTA, 0.5% SDS, 10 mM Tris pH 7.4) and vortexed to remove cells. An equal volume of acid phenol (pH 4.5) was added and the mixture was incubated at 65°C for 1 hour with frequent mixing. The entire extraction was transferred to a phase-lock gel tube (5Prime Inc., Gaithersburg, MD) and centrifuged according to the manufacturer's instructions. The aqueous layer was extracted twice more with chloroform∶isoamyl alcohol (24∶1) and precipitated with ethanol. Pellets were dried and dissolved in RNase free water, treated with 0.1U/µl RQ1 RNase-free DNase at 37°C for 1 hour (Promega, Madison WI), then further purified using the Qiagen RNeasy Mini kit. RNA quality was assessed on agarose denaturing gels as well as using a Bioanalyzer (Agilent Technologies) and quantified spectrophotometrically.

### Array design

Microarrays were produced using full-length open reading frame PCR products generated with the Sigma-Genosys ORFmers primer set and reaction conditions and cycling parameters recommended by the manufacturer (Sigma-Genosys, The Woodlands, TX). This set contains primer pairs for all 4290 known and hypothetical ORFs in *E. coli* K12 MG1655. PCR reactions were repeated and pooled as necessary to obtain at least 3 µg of DNA and pooled reactions were ethanol precipitated, resuspended and further purified using a Qiagen MinElute96 UF PCR purification kit. Purified PCR products were run on agarose gels for quantification and to verify PCR product size. 192 PCR products were excluded because they were either the wrong size, produced multiple product bands, or failed to amplify after repeated attempts. An additional 19 ORFs amplified poorly and consequently were spotted at lower levels on the arrays, but were retained in the analyses (see Table S4). Products were standardized to each contain 2 µg (except as noted in Table S4), dried, and dissolved in 10 µl 3× SSC for printing. Arrays were printed onto Corning Gaps II aminosilane-coated slides using a 48-pin Stanford-UCSF style arrayer at the Stanford Functional Genomics Facility (Stanford, CA).

### Array-Based Comparative Genome Hybridization (a-CGH) and expression profiling

Microarray expression profiling and CGH were performed using protocols developed at the J. Craig Venter Institute (http://pfgrc.jcvi.org/index.php/microarray/protocols.html) with the following modifications. For a-CGH, 5 µg of genomic DNA was sonicated to an average fragment length of 2–5 kb using a Branson Digital Sonifier at 11% amplitude for 1.1 seconds and a final concentration of 0.5 mM, and 1∶1 aa-dUTP∶dTTP labeling mixture was used in the Klenow reaction. For expression profiling, 20 µg of total RNA was reverse transcribed using 9 µg of random hexamer and 0.83 mM 1:1 aa-dUTP:dTTP labeling mixture. Slides were blocked (using 5× SSC, 0.1% SDS, 1% Roche Blocking Reagent) prior to hybridization as described (http://www.genomics.princeton.edu/dunham/MDhomemadeDNA.pdf) (Roche Applied Science, Mannheim, Germany). Hybridized arrays were scanned using an Axon 4000B scanner (Molecular Devices, Sunnyvale, CA).

### Quantitative RT–PCR

qRT-PCR was performed using the Step-One Plus Real-Time PCR System (Applied Biosystems (ABI), Foster City, CA). Primers and probes were designed using the default parameters with Primer Express 3.0 and purchased from Integrated DNA Technologies (IDT, Coralville, IA). A 2 µg aliquot of total RNA was treated with RNAse-free DNAse to remove residual DNA and subsequently reverse transcribed using the ABI High Capacity cDNA Reverse Transcription Kit, after which 1 µl of cDNA was added to 1X TaqMan Gene Expression Master Mix containing 900 nM each primer and 250 nM probe and cycled using the universal cycling program for the StepOne system. Relative amounts of each transcript were calculated using the ΔΔC_t_ method using *mdaB* as an endogenous control [109]. The sequences of the primers and probes used are shown in Table S5.

### Image processing and statistical methods

a-CGH images were processed using a combination of GenePix Pro 6.0, the TIGR TM4 software suite available at (www.tm4.org), and Microsoft Excel [110]. Image analysis and spot filtering was done in GenePix and a-CGH spots were considered acceptable if they: (1) passed the default flag conditions imposed by the software during spot finding; (2) had an intensity∶background ratio >1.5 and overall intensity >350 in the reference channel; and (3) had an intensity∶background ratio >1.0 in the experimental channel. GenePix files were converted to TIGR MEV format using Express Converter. Ratios were normalized using total intensity normalization and replicate spots were averaged using TIGR MIDAS software. Results were viewed using Caryoscope 3.0.9 (caryoscope.stanford.edu). One a-CGH comparison was performed for each experimental isolate using the ancestor JA122 as the reference genome.

For transcriptional profiling, spots were considered acceptable if the regression R^2^ was >0.6, or the sum of the median intensities for each channel minus the median background was >500. Spots that contained saturated pixels in both channels were excluded from the analysis, but spots that were saturated in only one channel were flagged and retained. Again, GenePix results were converted to TIGR MEV format using Express Converter and ratios normalized and averaged using TIGR MIDAS. Results were viewed and analyzed using TIGR MeV. Three comparisons, including one dye-flip pair, were performed for each biological replicate for a total of nine comparisons for each strain with the exception of CV116 which only had eight comparisons due to a technical failure. Genes that did not have acceptable spots for 2 out of the 3 biological replicates were excluded from downstream analyses. For each biological replicate, reference RNA was prepared from independent JA122 as described above.

Significance Analysis of Microarrays [111] (SAM) was used to examine expression differences between strains using a multi-class comparison consisting of four groups. Similarities among strains were identified using one-class SAM and differences between the strains were examined using a 4-class SAM. δ cutoffs were either (1) assigned visually, a strategy in which the tuning parameter (δ) was adjusted manually to reflect a natural break in the plot of observed vs. expected d-values from a line with slope = 1 (which resulted in a FDR of 0%), or (2) set at the 0% FDR threshold (i.e. the highest δ value that gave a median false discovery rate of 0%). In all cases, these settings resulted in q-values of 0. The default settings for all other parameters were retained. The average (mean) log_2_ ratios for biological and technical replicates were calculated after SAM analysis using Microsoft Excel.

Pair-wise Pearson correlation coefficients between array and qRT-PCR expression data were calculated as in [112] using Microsoft Excel.

### Regulon comparisons

Trancription unit, regulon and operon information was collated from the EcoCyc Database at http://www.ecocyc.org [63]. Predicted regulatory binding site information was obtained via TractorDB (http://www.tractor.lncc.br) [64].

### Data archiving

Data are available through the NIH GEO database under accession number GSE17314.

## Supporting Information

Figure S1 REP-PCR BoxA1R fingerprints of the terminal chemostat isolates are indistinguishable from those of the ancestor, JA122.
(0.18 MB PDF)

Figure S2 Global transcriptional response of evolved clones. Hierarchical clustering was performed on the averaged transcriptional profiles for each adaptive clone relative to its common ancestor, JA122. Adaptive clones and their ancestor were grown to steady state in chemostat monoculture. Evolved clones are shown as columns with each row representing a single gene. On average, about 7% (279 genes) of the transcriptome showed a two-fold or greater expression change in the adapted clones relative to their ancestor. Of these, decreases in transcript abundance were observed more often than increases (168 versus 111 genes).
(0.11 MB PDF)

Figure S3 Overview of central metabolic transcriptional response. The transcriptional response of several genes in glucose uptake, glycolysis, the pentose phosphate pathway, mixed acid fermentation, aromatic amino acid biosynthesis, and the tricarboxylic acid cycle were overlaid on a map of central metabolism. Red boxes or shading indicate that the gene was up-regulated while green denotes down-regulation. Blue boxes indicate that the gene is differently expressed among the four evolved clones. Yellow boxes denote gene deletion in CV103. Unshaded genes did not have significant transcript level differences compared to the ancestor. Average log2 evolved/ancestor values for differentially expressed genes are displayed in the table at the bottom.
(0.21 MB PDF)

Figure S4 qRT-PCR results for lamB, flgB, and acs. Mean expression vectors for log2 ratios were plotted for the microarray and qRT-PCR vlues for each gene and the Pearson correlation coefficient between techniques was calculated as in Larkin et al. 2005 [112]. The correlation coefficient for all three genes were high (0.78–0.99) indicating a strong correspondence between microarray and qRT-PCR transcript measurements.
(0.21 MB PDF)

Table S1 Top 90 significant genes by 1-class SAM for evolved isolates grown individually.
(0.13 MB PDF)

Table S2 Top 91 significant genes by 4-class SAM for evolved isolates grown individually.
(0.20 MB PDF)

Table S3 Sequencing primers.
(0.13 MB PDF)

Table S4 Failed and low-concentration PCR reactions.
(0.15 MB PDF)

Table S5 Primers used for qRT-PCR.
(0.12 MB PDF)
