# Supplementary material for: E Unibus Plurum: Genomic Analysis of an Experimentally Evolved Polymorphism in Escherichia coli
Source: PLoS Genet. 2009 Nov 6;5(11):e1000713. doi: 10.1371/journal.pgen.1000713 (PMC2763269; doi:10.1371/journal.pgen.1000713)
Supplement: Figure S1 — REP-PCR BoxA1R fingerprints of the terminal chemostat isolates are indistinguishable from those of the ancestor, JA122. (0.18 MB PDF) [file pgen.1000713.s001.pdf]

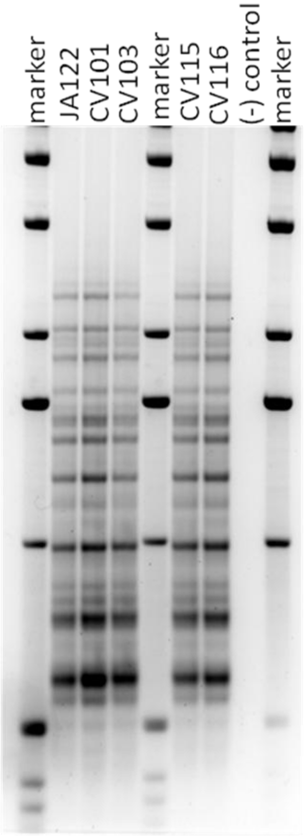

Supplementary Figure 1. REP-PCR BoxA1R fingerprints of the terminal chemostat isolates are indistinguishable from those of the ancestor, JA122.
