# Supplementary material for: E Unibus Plurum: Genomic Analysis of an Experimentally Evolved Polymorphism in Escherichia coli
Source: PLoS Genet. 2009 Nov 6;5(11):e1000713. doi: 10.1371/journal.pgen.1000713 (PMC2763269; doi:10.1371/journal.pgen.1000713)
Supplement: Figure S2 — Global transcriptional response of evolved clones. Hierarchical clustering was performed on the averaged transcriptional profiles for each adaptive clone relative to its common ancestor, JA122. Adaptive clones and their ancestor were grown to steady state in chemostat monoculture. Evolved clones are shown as columns with each row representing a single gene. On average, about 7% (279 genes) of the transcriptome showed a two-fold or greater expression change in the adapted clones relative to their ancestor. Of these, decreases in transcript abundance were observed more often than increases (168 versus 111 genes). (0.11 MB PDF) [file pgen.1000713.s002.pdf]

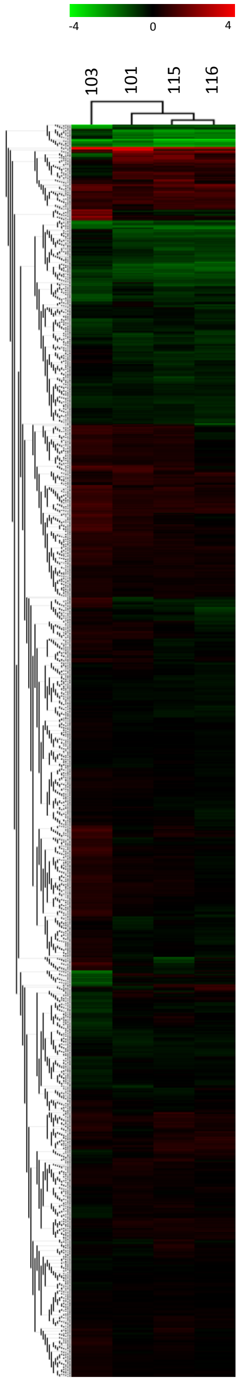

Supplementary Figure 2

Supplementary Figure 2. **Global transcriptional response of evolved clones.** Hierarchical clustering was performed on the averaged transcriptional profiles for each adaptive clone relative to its common ancestor, JA122. Adaptive clones and their ancestor were grown to steady state in chemostat monoculture. Evolved clones are shown as columns with each row representing a single gene. On average, about 7% (279 genes) of the transcriptome showed a two-fold or greater expression change in the adapted clones relative to their ancestor. Of these, decreases in transcript abundance were observed more often than increases (168 versus 111 genes).
