# Supplementary material for: E Unibus Plurum: Genomic Analysis of an Experimentally Evolved Polymorphism in Escherichia coli
Source: PLoS Genet. 2009 Nov 6;5(11):e1000713. doi: 10.1371/journal.pgen.1000713 (PMC2763269; doi:10.1371/journal.pgen.1000713)
Supplement: Figure S3 — Overview of central metabolic transcriptional response. The transcriptional response of several genes in glucose uptake, glycolysis, the pentose phosphate pathway, mixed acid fermentation, aromatic amino acid biosynthesis, and the tricarboxylic acid cycle were overlaid on a map of central metabolism. Red boxes or shading indicate that the gene was up-regulated while green denotes down-regulation. Blue boxes indicate that the gene is differently expressed among the four evolved clones. Yellow boxes denote gene deletion in CV103. Unshaded genes did not have significant transcript level differences compared to the ancestor. Average log2 evolved/ancestor values for differentially expressed genes are displayed in the table at the bottom. (0.21 MB PDF) [file pgen.1000713.s003.pdf]

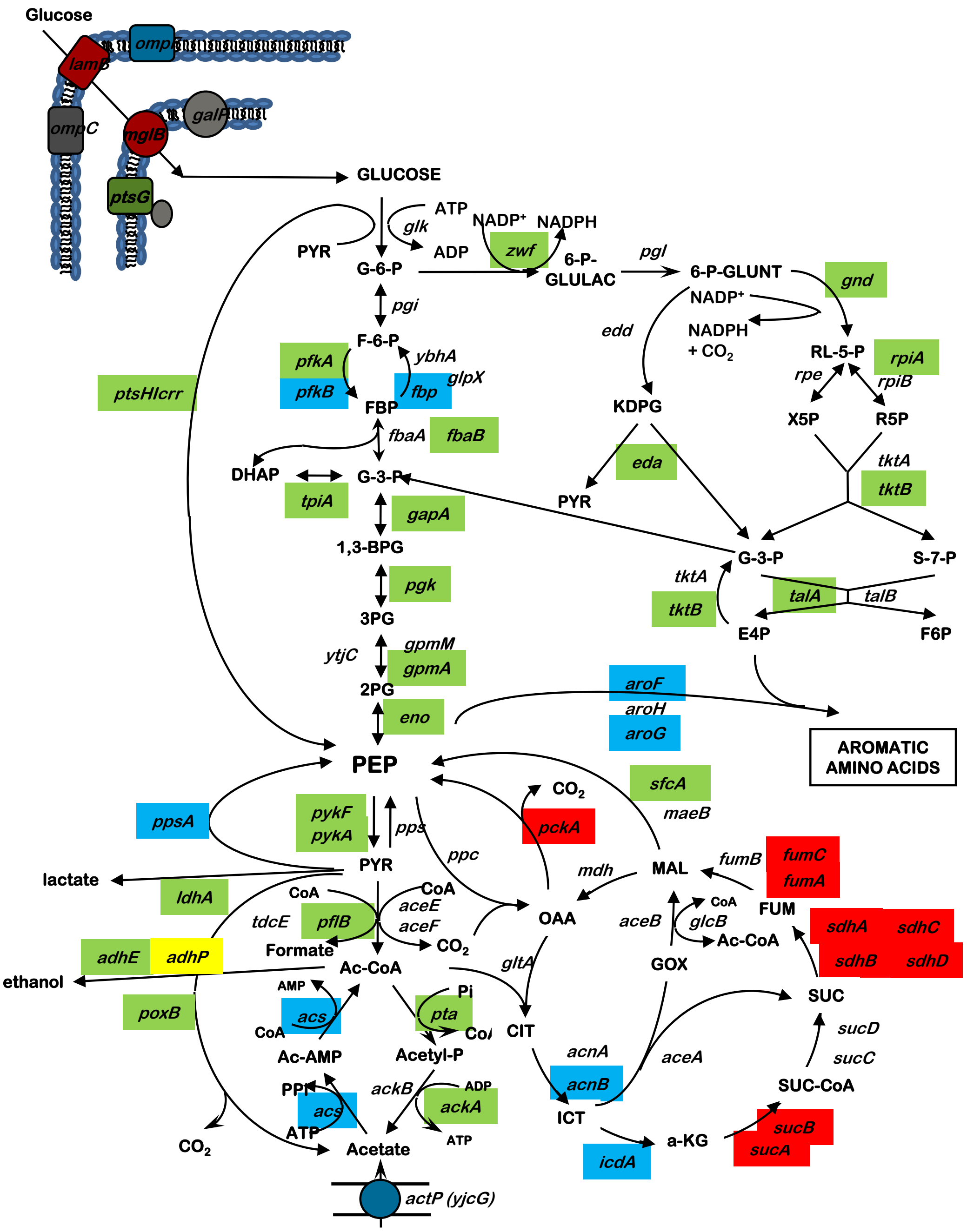

	101	103	115	116
acnB	1.13	-0.20	1.00	0.87
ppsA	0.02	-1.84	0.29	0.43
aroF	-0.56	0.86	-0.52	-0.63
aroG	0.14	-1.48	0.17	0.15
icdA	0.67	-0.42	0.92	0.81
pfkB	-1.47	-0.22	-1.20	-1.09
fbp	0.16	-0.67	-0.10	0.14

Supplementary Figure 3. Overview of central metabolic transcriptional response. The transcriptional response of several genes in glucose uptake, glycolysis, the pentose phosphate pathway, mixed acid fermentation, aromatic amino acid biosynthesis and the tricarboxylic acid cycle were overlaid on a map of central metabolism. Red boxes or shading indicate that the gene was up-regulated while green denotes down-regulation. Blue boxes indicate that the gene is differently expressed among the four evolved clones. Yellow boxes denote gene deletion in CV103. Unshaded genes did not have significant transcript level differences compared to the ancestor. Average log_2 evolved/ancestor values for differentially expressed genes are displayed in the table at the bottom.
