# Supplementary material for: E Unibus Plurum: Genomic Analysis of an Experimentally Evolved Polymorphism in Escherichia coli
Source: PLoS Genet. 2009 Nov 6;5(11):e1000713. doi: 10.1371/journal.pgen.1000713 (PMC2763269; doi:10.1371/journal.pgen.1000713)
Supplement: Figure S4 — qRT-PCR results for lamB, flgB, and acs. Mean expression vectors for log2 ratios were plotted for the microarray and qRT-PCR vlues for each gene and the Pearson correlation coefficient between techniques was calculated as in Larkin et al. 2005 [112]. The correlation coefficient for all three genes were high (0.78–0.99) indicating a strong correspondence between microarray and qRT-PCR transcript measurements. (0.21 MB PDF) [file pgen.1000713.s004.pdf]

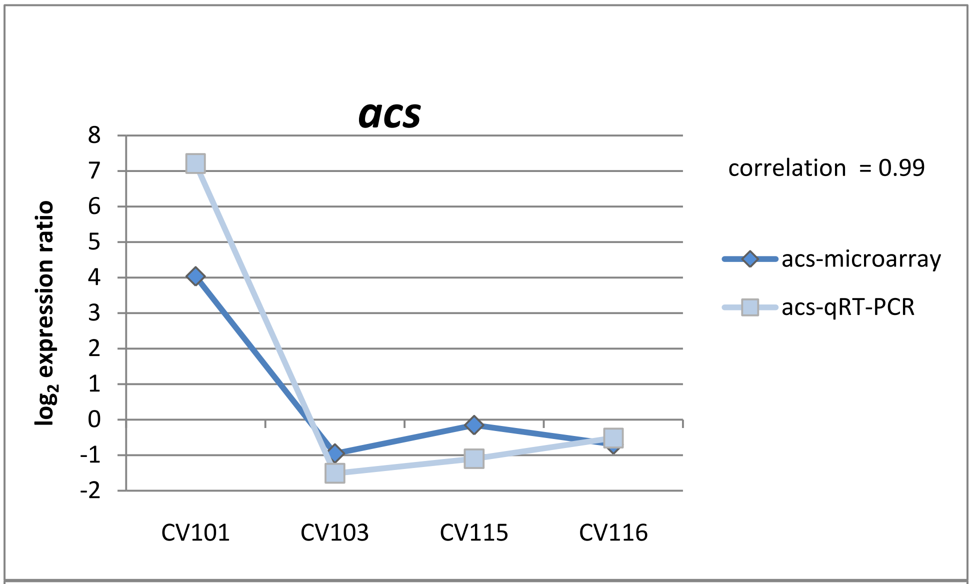
acs
8
7
6
5
4
3
2
1
0
-1
-2
log2 expression ratio
CV101
CV103
CV115
CV116
correlation = 0.99
acs-microarray
acs-qRT-PCR

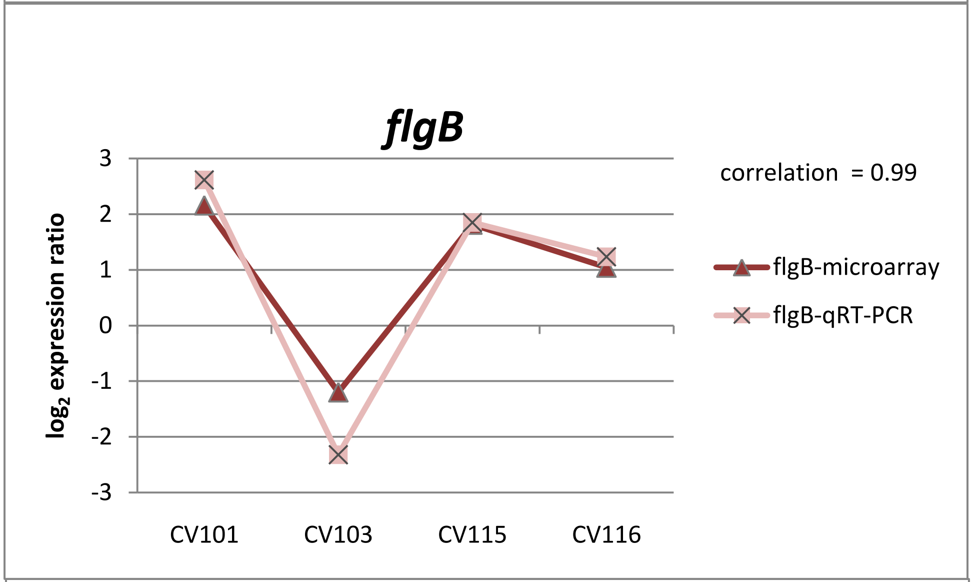
flgB
3
2
1
0
-1
-2
-3
log2 expression ratio
CV101
CV103
CV115
CV116
correlation = 0.99
flgB-microarray
flgB-qRT-PCR

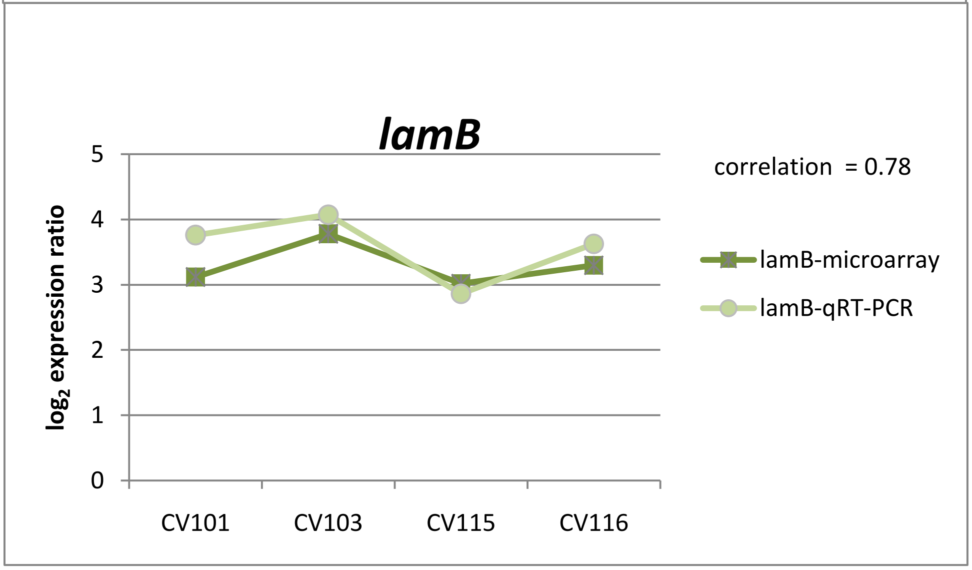
lamB
5
4
3
2
1
0
log2 expression ratio
CV101
CV103
CV115
CV116
correlation = 0.78
lamB-microarray
lamB-qRT-PCR

Supplementary Figure 4. qRT-PCR results for lamB, flgB, and acs. Mean expression vectors for log2 ratios were plotted for the microarray and qRT-PCR vlues for each gene and the Pearson correlation coefficient between techniques was calculated as in Larkin et al. 2005 [112]. The correlation coefficient for all three genes were high (0.78-0.99) indicating a strong correspondence between microarray and qRT-PCR transcript measurements.
