# Supplementary material for: E Unibus Plurum: Genomic Analysis of an Experimentally Evolved Polymorphism in Escherichia coli
Source: PLoS Genet. 2009 Nov 6;5(11):e1000713. doi: 10.1371/journal.pgen.1000713 (PMC2763269; doi:10.1371/journal.pgen.1000713)
Supplement: Table S1 — Top 90 significant genes by 1-class SAM for evolved isolates grown individually. (0.13 MB PDF) [file pgen.1000713.s005.pdf]

Supplementary Table 1. Top 90 significant genes by 1-class SAM for evolved isolates grown individually.

ID	gene	Log_2 CV101/ JA122	Log_2 CV103/ JA122	Log_2 CV115/ JA122	Log_2 CV116/ JA122	gene product	Transcription Unit	MultiFun category
b4036	***lamB***	3.6	5.1	2.9	2.2	maltose outer membrane porin (maltoporin)	malK-lamB-malM (malKp)	transport; (The Outer Membrane Porin (OMP) Functional Superfamily); The Sugar Porin (SP) Family
b2148	***mglC***	3.5	3.1	2.6	2.5	membrane component of an ABC superfamily methyl-galactoside transporter	mglBAC (mglBp)	metabolism; carbon utilization; transport; The ATP-binding Cassette (ABC) Superfamily
b3902	***rhaD***	3.4	3.4	2.7	2.7	rhamnulose-1-phosphate aldolase	rhaBAD (rhaBp)	Metabolism; carbon utilization;
b2149	***mglA***	4.5	3.9	3.6	3.3	fused methyl-galactoside transporter subunits of ABC superfamily: ATP-binding components	mglBAC (mglBp)	metabolism; carbon utilization; The ATP-binding Cassette (ABC) Superfamily
b2150	***mglB***	4.1	4.1	3.6	2.7	periplasmic-binding component of an ABC superfamily methyl-galactoside transporter	mglBAC (mglBp)	Metabolism; carbon utilization; chaperoning, folding, transport; Primary Active Transporters; The ATP-binding Cassette (ABC) Superfamily
b1804	***rnd***	0.6	0.6	0.6	0.7	ribonuclease D	rnd	metabolism; macromolecule degradation; information transfer; RNA modification
b0589	***fepG***	1.0	1.0	0.7	1.0	membrane component of an ABC superfamily iron-enterobactin transporter	fepDGC (fepDp1)	transport; The ATP-binding Cassette (ABC) Superfamily
b3505	***insH-11***	0.7	1.0	1.2	1.2	IS5 transposase	insH-11	Extrachromosomal; transposon related
b3218	***insH-10***	0.7	0.9	1.1	1.1	IS5 transposase	insH-10	Extrachromosomal; transposon related
b0656	***insH-3***	0.6	0.9	1.0	1.0	IS5 transposase	insH-3	Extrachromosomal; transposon related
b0552	***insH-2***	0.7	0.8	1.0	1.1	IS5 transposase	insH-2	Extrachromosomal; prophage genes;transposon related
b2192	***insH-8***	0.6	0.9	1.0	1.1	IS5 transposase	insH-8	Extrachromosomal; transposon related
b1370	***insH-5***	0.7	0.9	1.0	1.1	IS5 transposase	insH-5	Extrachromosomal; prophage genes; transposon related
b1331	***insH-4***	0.6	0.9	1.0	1.1	IS5 transposase	insH-4	Extrachromosomal; transposon related
b2030	***insH-7***	0.6	0.9	1.1	1.1	IS5 transposase	insH-7	Extrachromosomal; transposon related
b2982	***insH-9***	0.6	0.9	1.1	1.2	IS5 transposase	insH-9	Extrachromosomal; transposon related
b0259	***insH-1***	0.7	0.9	1.1	1.1	IS5 transposase	insH-1	Extrachromosomal; prophage genes; transposon related

b1994	***insH-6***	0.6	0.9	1.1	1.1	IS5 transposase	insH-6	Extrachromosomal; transposon related
b2289	***lrhA***	0.9	0.9	1.1	1.1	DNA-binding transcriptional repressor of flagellar, motility and chemotaxis genes	lrhA	metabolism; energy metabolism, aerobic respiration, regulation; transcriptional level; repressor
b2147	***yeiA***	1.4	1.7	1.6	1.4	predicted oxidoreductase	yeiAT (yeiTp)	metabolism; central intermediary metabolism; unassigned reversible reactions
b2151	***galS***	1.9	2.0	2.0	2.2	DNA-binding transcriptional repressor	galS	metabolism; carbon utilization; regulation; transcriptional level; repressor
b1042	***csgA***	-2.9	-2.7	-4.0	-4.3	cryptic curlin major subunit	csgBAC (csgBp)	metabolism; macromolecules (cellular constituent) biosynthesis; glycoprotein, cell structure; pilus,
b1923	***fliC***	-2.6	-2.7	-3.5	-3.8	flagellar filament structural protein (flagellin)	fliC	metabolism; macromolecules (cellular constituent) biosynthesis; flagella
b0897	***ycaC***	-2.9	-1.8	-3.6	-3.6	predicted hydrolase	ycaC	Metabolism; carbon utilization; amino acids
b1444	***ydcW***	-1.4	-2.6	-3.3	-3.3	medium chain aldehyde dehydrogenase	ydcW	
b3555	***yiaG***	-2.0	-0.6	-2.4	-2.4	predicted transcriptional regulator	yiaG	regulation; transcriptional level
b2665	***ygaU***	-2.5	-1.0	-2.7	-2.6	predicted protein	ygaU	
b2080	***yegP***	-2.4	-1.1	-2.6	-2.4	predicted protein	yegP	
b1896	***otsA***	-2.4	-0.7	-2.6	-2.4	trehalose-6-phosphate synthase	otsBA (otsBp)	metabolism; central intermediary metabolism; glucose metabolism,
b1051	***msyB***	-2.3	-0.8	-2.4	-2.3	predicted protein	msyB	transport; substrate; protein
b0812	***dps***	-2.8	-2.2	-2.9	-2.4	Fe-binding and storage protein	dps	Information transfer; protein related; cell processes; adaptation to stress; starvation response
b4376	***osmY***	-2.3	-1.8	-2.8	-3.0	periplasmic protein	osmY	Cell processes; adaptation to stress; osmotic pressure
b3447	***ggt***	-2.2	-1.9	-2.9	-2.9	gamma-glutamyltranspeptidase	ggt	metabolism; macromolecules (cellular constituent) biosynthesis; thioredoxin, glutaredoxin
b1258	***yciF***	-2.6	-1.5	-2.8	-2.6	conserved protein	yciGFE (yciGp)	
b1885	***tap***	-1.5	-2.0	-2.8	-2.9	methyl-accepting protein IV	tar-tap-cheRBYZ (tarp)	regulation; posttranscriptional; cell processes; motility (incl.
b1443	***ydcV***	-1.4	-2.4	-2.5	-2.8	membrane component of an ABC superfamily predicted spermidine/putrescine transporter	ydcSTUV	transport; The ATP-binding Cassette (ABC) Superfamily
b1040	***csgD***	-1.7	-1.8	-2.3	-2.6	DNA-binding transcriptional regulator of adhesion determinants	csgDEFG (csgDp2)	Information transfer; RNA related; transcription related,activator

b1967	***hchA***	-1.9	-1.9	-2.8	-2.0	Hsp31 molecular chaperone	hchA	
b2924	***mscS***	-2.0	-1.7	-1.9	-2.1	component of the MscS mechanosensitiv e channel	mscS	transport; Channel-type Transporters; The Small Conductance Mechanosensitive Ion Channel (MscS) Family
b1038	***csgF***	-1.8	-1.8	-2.1	-2.0	predicted transport protein	csgDEFG (csgDp2)	transport; Putative uncharacterized transport protein, cell structure; pilus, curli subunit
b1480	***sra***	-2.0	-1.7	-2.3	-2.3	30S ribosomal subunit protein S22	sraA (sraAp)	Information transfer; protein related; ribosomal proteins
b1482	***osmC***	-2.1	-1.9	-2.2	-2.1	osmotically inducible, stress-inducible membrane protein	osmC	Cell processes; adaptation to stress; osmotic pressure
b2465	***tktB***	-1.9	-1.3	-2.5	-2.3	transketolase 2, thiamin-binding	tktB	metabolism; carbon utilization; central intermediary metabolism; pentose phosphate shunt, non-oxidative branch,;nucleotide and nucleoside conversions
b2266	***elaB***	-1.8	-1.3	-2.3	-2.2	conserved protein	elaB	
b2097	***fbaB***	-1.8	-1.2	-2.3	-2.1	fructose-bisphosphate aldolase class I	fbaB	metabolism; energy metabolism, carbon; glycolysis
b1004	***wrbA***	-2.0	-1.4	-2.3	-2.0	predicted flavoprotein in Trp regulation	wrbA-yccJ (wrbAp)	Metabolism; building block biosynthesis; amino acids; tryptophan
b1732	***katE***	-1.9	-1.5	-2.2	-2.1	hydroperoxidase HPII(III) (catalase)	katE	Cell processes; protection; detoxification (xenobiotic metabolism)
b0871	***poxB***	-1.6	-2.7	-2.0	-1.9	pyruvate dehydrogenase (pyruvate oxidase), thiamin-dependent, FAD-binding	ltaE-poxB-ybjT	metabolism; carbon utilization; central intermediary metabolism; pyruvate oxidation
b1478	***adhP***	-1.3	-2.2	-1.8	-1.7	alcohol dehydrogenase, 1-propanol preferring	adhP	metabolism; energy metabolism, carbon; anaerobic respiration
b1037	***csgG***	-1.6	-1.9	-1.7	-1.4	outer membrane channel lipoprotein	csgDEFG (csgDp2)	transport; Putative uncharacterized transport protein, cell structure; pilus, curli subunit
b1431	***ydcL***	-1.5	-1.5	-1.6	-1.8	predicted lipoprotein	ydcL	
b1490	***yddV***	-1.5	-1.5	-1.5	-1.8	predicted diguanylate cyclase	yddV-dos	
b1197	***treA***	-1.6	-1.3	-1.8	-1.9	periplasmic trehalase	treA	metabolism; central intermediary metabolism; adaptation to stress; osmotic pressure
b1101	***ptsG***	-1.8	-1.4	-1.7	-1.4	PTS system glucose-specific IICB component	ptsG	Metabolism; carbon utilization; regulation; type of regulation; posttranscriptional; transport
b1259	***yciG***	-1.7	-1.5	-1.8	-1.5	predicted protein	yciGFE (yciGp)	
b0753	***ybgS***	-1.9	-1.2	-1.9	-1.7	conserved protein	ybgS	
b3515	***gadW***	-1.6	-1.3	-2.1	-1.7	DNA-binding transcriptional activator	gadXW (gadXp)	Information transfer; RNA related; transcription related, regulation; activator
b1710	***btuE***	-1.5	-1.1	-2.0	-1.8	predicted glutathione	btuCED	Metabolism; building block biosynthesis; cobalamin (Vitamin

						peroxidase		B12), transport; Primary Active Transporters; The ATP-binding Cassette (ABC) Superfamily
b1810	***yoaC***	-1.8	-0.9	-1.8	-2.1	predicted protein	yoaC	
b1050	***yceK***	-1.9	-0.9	-2.4	-1.8	predicted lipoprotein	yceK	
b2464	***talA***	-1.9	-1.0	-2.1	-1.8	transaldolase A	talA	metabolism; central intermediary metabolism; pentose phosphate shunt, non-oxidative branch
b0453	***ybaY***	-1.9	-1.0	-2.1	-2.0	predicted outer membrane lipoprotein	ybaY	metabolism; macromolecules biosynthesis; lipoprotein; glycoprotein
b1836	***yebV***	-1.4	-0.7	-2.2	-1.9	predicted protein	yebV	
b1064	***grxB***	-1.6	-1.3	-1.6	-1.0	glutaredoxin 2 (Grx2)	grxB	metabolism; macromolecules (cellular constituent) biosynthesis; large molecule carriers; thioredoxin, glutaredoxin
b1003	***yccJ***	-1.3	-1.1	-1.7	-1.4	predicted protein	wrbA-yccJ (wrbAp)	
b1241	***adhE***	-1.4	-0.7	-1.2	-1.6	fused acetaldehyde-CoA dehydrogenase and iron-dependent alcohol dehydrogenase and pyruvate-formate lyase deactivase	adhE	metabolism; energy metabolism, carbon; fermentation
b1709	***btuD***	-1.1	-0.6	-1.7	-1.3	vitamin B12 transporter subunit : ATP-binding component of ABC superfamily	btuCED	Metabolism; building block biosynthesis; cobalamin (Vitamin B12), transport; The ATP-binding Cassette (ABC) Superfamily
b1661	***cfa***	-1.5	-0.6	-1.6	-1.3	cyclopropane fatty acyl phospholipid synthase	cfa	Metabolism; building block biosynthesis; fatty acid and phosphatidic acid
b2594	***rluD***	-0.4	-0.5	-0.6	-0.6	23S rRNA pseudouridine synthase	rluD-yfiH	Information transfer; RNA related; RNA modification
b1049	***mdo H***	-0.4	-0.7	-0.8	-0.6	glycosyl transferase	mdoGH (mdoGp1)	Cell processes; adaptation to stress; osmotic pressure
b1641	***slyB***	-0.8	-1.3	-0.8	-0.7	outer membrane lipoprotein	slyB	Cell structure; membrane
b4384	***deoD***	-0.7	-0.9	-0.9	-0.7	purine-nucleoside phosphorylase	deoCABD (deoCp1)	metabolism; central intermediary metabolism; nucleotide and nucleoside conversions
b0708	***phr***	-0.8	-0.9	-0.9	-0.8	deoxyribodipyrimidine photolyase	ybgA-phr (ybgAp1)	Metabolism; building block biosynthesis; riboflavin (Vitamin B2), FAD, FMN, information transfer; DNA related; DNA repair
b3401	***hslO***	-0.8	-0.7	-0.8	-0.9	heat shock protein Hsp33	hslR-hslO (hslRp)	Information transfer; protein related; chaperoning, folding
b2415	***ptsH***	-0.9	-0.6	-0.8	-0.9	phosphohistidinoprotein-hexose phosphotransferase component of PTS system (Hpr)	ptsHI-crr (ptsHp1)	metabolism; carbon utilization; Group Translocators; The Phosphotransferase System HPr (HPr) Family, transport; substrate; sugar
b3919	***tpiA***	-0.9	-0.9	-1.0	-1.1	triosephosphate isomerase	tpiA	metabolism; energy metabolism, carbon; glycolysis

b1380	***ldhA***	-1.0	-0.8	-0.8	-1.0	fermentative D-lactate dehydrogenase, NAD-dependent	ldhA	metabolism; energy metabolism, carbon; fermentation
b4383	***deoB***	-1.1	-1.2	-1.2	-1.1	phosphopentom utase	deoCABD (deoCp1)	metabolism; central intermediary metabolism; nucleotide and nucleoside conversions
b0903	***pflB***	-0.6	-0.9	-1.1	-1.1	pyruvate formate lyase I	focA-pflB (focAp1)	metabolism; energy metabolism, carbon; anaerobic respiration, carbon utilization; central intermediary metabolism; threonine catabolism
b1039	***csgE***	-1.0	-0.8	-1.5	-1.4	predicted transport protein	csgDEFG (csgDp2)	transport; Transporters of Unknown Classification; Putative uncharacterized transport protein, cell structure; pilus,curli
b1957	***yodC***	-1.0	-0.9	-1.4	-1.2	predicted protein	yodC	
b0904	***focA***	-1.0	-0.7	-1.4	-1.2	formate transporter	focA-pflB (focAp1)	metabolism; carbon utilization; transport; The Formate-Nitrite Transporter (FNT) Family
b2687	***luxS***	-1.1	-0.8	-1.3	-1.2	S-ribosylhomocyst eine lyase	luxS	regulation; transcriptional level; complex regulation; quorum sensing
b3024	***ygiW***	-1.1	-0.8	-1.3	-1.2	conserved protein	ygiW	
b2779	***eno***	-1.0	-1.0	-1.2	-1.4	enolase	eno-pyrG (pyrGp)	metabolism; energy metabolism, carbon; glycolysis; anaerobic respiration; gluconeogenesis
b1795	***yeaQ***	-1.1	-0.9	-1.1	-1.1	conserved inner membrane protein	yeaQ	
b0707	***ybgA***	-1.2	-0.7	-1.2	-1.0	conserved protein	ybgA-phr (ybgAp1)	
b3336	***bfr***	-1.1	-0.7	-1.1	-1.0	bacterioferritin, iron storage and detoxification protein	bfd-bfr	Cell processes; adaptation to stress; Fe aquisition
b0965	***yccU***	-0.9	-0.6	-1.3	-1.0	predicted CoA-binding protein	yccU	
b2417	***crr***	-0.9	-0.5	-1.1	-1.1	glucose-specific enzyme IIA component of PTS	ptsHI-crr (ptsHp1)	metabolism; carbon utilization; The PTS Fructose-Mannitol (Fru) Family, transport; substrate; D-glucose/trehalose
