# Supplementary material for: E Unibus Plurum: Genomic Analysis of an Experimentally Evolved Polymorphism in Escherichia coli
Source: PLoS Genet. 2009 Nov 6;5(11):e1000713. doi: 10.1371/journal.pgen.1000713 (PMC2763269; doi:10.1371/journal.pgen.1000713)
Supplement: Table S2 — Top 91 significant genes by 4-class SAM for evolved isolates grown individually. (0.20 MB PDF) [file pgen.1000713.s006.pdf]

Supplementary Table 2. Top 91 significant genes by 4-class SAM for evolved isolates grown individually.

ID	Gene	mean log_2 CV101 /JA122	mean log_2 CV101 /JA122	mean log_2 CV101 /JA122	mean log_2 CV101 /JA122	Gene product	Transcription unit	MultiFun description
b4484	***cpxP***	0.0	1.7	-0.8	0.0	regulator of Cpx response; possible chaperone involved in extra-cytoplasmic stress resistance	cpxP	Cell processes; adaptations; regulation; 2-component regulatory system
b1874	***cutC***	-0.4	1.8	-1.0	-0.6	copper homeostasis protein	cutC	Cell processes; protection; detoxification (xenobiotic metabolism)
b1552	***cspI***	0.0	0.8	-0.1	-0.2	Qin prophage; cold shock protein	cspI	Prophage genes and phage related functions; extrachromosomal
b2630	***yfjN***	-0.4	0.8	0.0	-0.1	CP4-57 prophage; RNase LS	rnlA-yfjO	Prophage genes and phage related functions; extrachromosomal
b4170	***mutL***	0.0	0.8	0.1	0.0	methyl-directed mismatch repair protein	yjeFE-amiB-mutL-miaA-hfq-hflXKC	Information transfer; DNA repair
b4171	***miaA***	-0.3	1.0	0.1	-0.4	δ-(2)-iso-pentenylpyro-phosphate tRNA-adenosine transferase	yjeFE-amiB-mutL-miaA-hfq-hflXKC	Information transfer; RNA related; RNA modification
b2597	***raiA***	-0.3	1.8	0.3	0.0	cold shock protein associated with 30S ribosomal subunit	raiA	Information transfer; protein related; translation, cell structure; ribosome; cold-shock protein
b1463	***nhoA***	-0.2	-2.0	-0.3	-0.3	N-hydroxyarylamine O-acetyltransferase	nhoA	Metabolism
b1466	***narW***	-0.5	-2.8	-1.1	-1.5	nitrate reductase 2 (NRZ), δ - subunit (assembly subunit)	narZYWV	Metabolism; Anaerobic respiration C & energy metabolism;, information transfer; protein related; chaperoning, folding
b1467	***narY***	-0.9	-3.0	-1.3	-1.5	nitrate reductase 2 (NRZ), β-subunit	narZYWV	Metabolism; Anaerobic respiration energy production/transport; *e*-acceptor;, carbon;, cell structure; membrane
b0430	***cyoC***	0.4	-0.8	1.2	0.7	cytochrome o ubiquinol oxidase subunit III	cyoABCDE	Metabolism; Aerobic respiration; C & energy metabolism; energy production/transport; e^- acceptor; Primary Active Transporters; Oxidoreduction-driven Active Transporters; H^+-translocating Cytochrome Oxidase (COX)
b0431	***cyoB***	0.4	-1.1	0.8	0.6	cytochrome o ubiquinol oxidase subunit I	cyoABCDE	Metabolism; Aerobic metabolism; e^- acceptor; C & energy metabolism; Primary Active Transporters; Oxido-reduction-driven Active Transporters; H^+-translocating Cytochrome Oxidase (COX)
b0432	***cyoA***	0.3	-1.2	0.7	0.9	cytochrome o ubiquinol oxidase subunit II	cyoABCDE	Metabolism; Aerobic respiration; e^- transport; C & energy metabolism; Primary Active Transporters; Oxido-reduction-driven Active Transporters; H^+-translocating Cyt oxidase (COX)
b0754	***aroG***	0.1	-1.5	0.2	0.2	3-deoxy-D-arabino-heptulosonate-7-phosphate synthase, phenylalanine repressible	aroG	Metabolism; Building block biosynthesis; amino acids; phenylalanine
b1073	***flgB***	1.3	-1.1	2.0	0.7	flagellar component of cell-proximal portion of basal-body rod	flgBCDEFGHIJ	Metabolism; Biosynthesis; flagellum macromolecules (cellular constituent) cell processes; motility (incl.

								chemotaxis, energytaxis, aerotaxis, redoxtaxis), cell structure;
b1074	***flgC***	1.3	-1.0	1.9	0.6	flagellar component of cell-proximal portion of basal-body rod	flgBCDEFGHIJ	Metabolism; Biosynthesis; flagellum macromolecules (cellular constituent), cell processes; motility (incl. chemotaxis, energytaxis, aerotaxis, redoxtaxis), cell structure
b1075	***flgD***	1.5	-1.4	2.0	0.7	flagellar hook assembly protein	flgBCDEFGHIJ	Metabolism; Biosynthesis; flagellum macromolecules (cellular constituent); cell processes; motility (incl. chemotaxis, energytaxis, aerotaxis, redoxtaxis), cell structure;
b1076	***flgE***	1.3	-1.5	2.0	0.7	flagellar hook protein	flgBCDEFGHIJ	Metabolism; Biosynthesis; flagellum macromolecules (cellular constituent); cell processes; motility (incl. chemotaxis, energytaxis, aerotaxis, redoxtaxis) , cell structure;
b1077	***flgF***	1.1	-1.1	1.4	0.5	flagellar component of cell-proximal portion of basal-body rod	flgBCDEFGHIJ	Metabolism; Biosynthesis; flagellum macromolecules (cellular constituent); cell processes; motility (incl. chemotaxis, energytaxis, aerotaxis, redoxtaxis), cell structure;
b1078	***flgG***	1.3	-1.0	1.5	0.7	flagellar component of cell-distal portion of basal-body rod	flgBCDEFGHIJ	Metabolism; Biosynthesis; flagellum macromolecules (cellular constituent), cell processes; motility (incl. chemotaxis, energytaxis, aerotaxis, redoxtaxis), cell structure;
b1079	***flgH***	1.1	-0.7	1.3	0.6	flagellar protein of basal-body outer-membrane L ring	flgBCDEFGHIJ	Metabolism; Biosynthesis; flagellum macromolecules (cellular constituent), cell processes; motility (incl. chemotaxis, energytaxis, aerotaxis, redoxtaxis) , cell structure; membrane, cell structure;
b1941	***fliI***	1.7	-0.6	2.0	0.7	flagellum-specific ATP synthase	fliFGHIJK (fliFp)	Metabolism; Biosynthesis; flagellum; C & energy metabolism; ATP-H^+ motive force interconversion, cell structure
b1944	***fliL***	0.9	-0.3	1.2	0.3	flagellar biosynthesis protein	fliLMNOPQR (fliLp1)	Metabolism; Biosynthesis; flagellum macromolecules (cellular constituent); cell processes; motility (incl. chemotaxis, energytaxis, aerotaxis, redoxtaxis) , cell structure;
b1945	***fliM***	1.6	-0.6	1.6	1.4	flagellar motor switching and energizing component	fliLMNOPQR (fliLp1)	Metabolism; Biosynthesis; flagellum macromolecules (cellular constituent), cell processes; motility (incl. chemotaxis, energytaxis, aerotaxis, redoxtaxis), cell structure;
b2553	***glnB***	0.2	-0.6	0.5	0.2	regulatory protein P-II for glutamine synthetase	glnB	Metabolism; Building block biosynthesis; amino acids; glutamine, information transfer; RNA related; transcriptional, regulation; post-transcriptional regulation; inhibition/activation of enzymes
b2378	***lpxP***	0.6	2.2	0.8	0.4	palmitoleoyl-acyl carrier protein (ACP)-dependent acyltransferase	lpxP	Metabolism; Biosynthesis; lipopolysaccharide; macro-molecules (cellular constituent) lipid A, cell processes; adaptation

								to thermal stress; cell structure; membrane, cell structure; surface antigens (ECA, O antigen of LPS)
b0750	***nadA***	-0.5	-1.9	-0.9	-0.8	quinolinate synthase, subunit A	nadA-pnuC	Metabolism; Building block biosynthesis; NAD+ carrier
b2476	***purC***	0.0	-1.5	-0.5	0.1	phosphoribosylaminoimidazole-succinocarboxamide synthetase	purC	Metabolism; Building block biosynthesis; purine biosynthesis
b0494	***tesA***	0.0	1.1	0.1	-0.1	multifunctional acyl-CoA thioesterase I and protease I and lysophospholipase L1	tesA	Metabolism; Building block biosynthesis; fatty acid & phosphatidic acid
b4069	***acs***	3.9	0.0	-1.5	-0.3	acetyl-CoA synthetase	acs-yjcHG	Metabolism; Building Block Biosynthesis; fatty acid and phosphatidic acid metabolism; acetate utilization; central intermediary metabolism;
b1896	***otsA***	-2.4	-0.7	-2.6	-2.4	trehalose-6-phosphate synthase	otsBA	Metabolism; central intermediary metabolism; glucose metabolism, cell processes; osmotic stress adaptation;
b1702	***pps***	0.0	-1.8	0.3	0.6	phosphoenolpyruvate synthase	pps	Metabolism; gluconeogenesis central intermediary metabolism
b2091	***gatD***	1.8	-0.5	1.3	0.8	galactitol-1-phosphate dehydrogenase, Zn-dependent and NAD(P)-binding	gatYZABCD	Metabolism; C utilization; galactitol
b2095	***gatZ***	2.0	-1.2	1.6	1.3	D-tagatose 1,6-bisphosphate aldolase 2, subunit	gatYZABCD	Metabolism; C utilization; tagatose
b2096	***gatY***	1.8	-1.4	1.2	1.0	D-tagatose 1,6-bisphosphate aldolase 2, catalytic subunit	gatYZABCD	Metabolism; C utilization; tagatose
b0161	***degP***	-0.4	1.9	-1.1	-0.9	serine endoprotease (protease Do), membrane-associated	degP	Metabolism; MacroMolecule Degradation; proteins/peptides/ glycopeptides, cell processes; adaptation to thermal stress;
b1844	***exoX***	0.0	1.1	0.3	0.2	DNA exonuclease X	exoX-yobB	Metabolism; macromolecule degradation; information transfer; DNA repair, DNA degradation
b2193	***narP***	-0.1	1.1	0.0	-0.1	DNA-binding response regulator in 2-component regulatory system with NarQ or NarX	narP	Transcriptional Regulation; Information Transfer; Transcriptional activator/repressor; C & energy metabolism; anaerobic respiration; RNA related;
b3461	***rpoH***	-0.2	0.8	-0.5	-0.7	RNA polymerase, σ^{32} (σ^{H}) factor	rpoH	Information transfer; Transcriptional Regulation; RNA related; □ factors, anti-□-factors; stimulon, cell processes; adaptation to stress; temperature extremes
b1663	***mdtK***	-0.5	0.4	-0.4	-0.4	multidrug efflux system transporter	mdtK	Transport; Multi Antimicrobial Extrusion (MATE) Family; Electrochemical potential driven transporters; Porters (Uni-, Sym- and Antiporters); cell processes; cell structure; protection; drug resistance/sensitivity, membrane
b0929	***ompF***	1.2	-1.7	1.3	0.0	outer membrane porin 1a (Ia;b;F)	ompF	Transport; □-barrel porins (Outer Membrane Porin (OMP) Functional Superfamily); Solute:Sodium Symporter (SSS) Family; Channel-type Transporters; General Bacterial Porin (GBP) Family, cell structure; hydrophilic molecule
b3540	***dppF***	0.1	1.6	1.1	1.0	dipeptide transporter; ATP-binding component of ABC superfamily	dppABCDF	Primary Active Transporters; ATP-binding Cassette (ABC) Superfamily + ABC-type Uptake Permeases; C utilization; amino acid transport; PP_i Bond (ATP, GTP, P2) Hydrolysis-driven Active Transporters; Metabolism
b3454	***livF***	0.1	-0.1	0.6	-1.5	leucine/isoleucine/valine transporter subunit; ATP-binding component of ABC superfamily	livKHMGF	Primary Active Transporters; Building block biosynthesis: (isoleucine/valine/leucine); amino acid

								transport/metabolism); PP_i Bond (ATP, GTP, P); ATP-binding Cassette (ABC) Superfamily + ABC-type
b3455	***livG***	0.1	-0.3	0.6	-1.6	leucine/isoleucine/valine transporter subunit; ATP-binding component of ABC superfamily	livKHMGF	Primary Active Transporters; building block biosynthesis: (isoleucine/valine/leucine); amino acid metabolism; PP_i Bond (ATP, GTP, P), ATP-binding Cassette (ABC) Superfamily + ABC-type
b1017	***efeU_2***	0.4	-0.9	0.6	1.1	C-terminal fragment of ferrous iron permease (pseudogene)	efeBO	Transporter pseudogene
b1018	***efeO***	0.2	-0.6	0.7	0.9	component of a tripartite ferrous iron transporter	efeBO	Transport
b3915	***fieF***	-0.3	0.6	-0.1	-0.2	zinc transporter	fieF	Transport; Cation Diffusion Facilitator (CDF) Family; Electro-chemical potential driven transporters; Porters (Uni-, Sym- and Antiporters); cell structure; membrane
b0763	***modA***	0.2	1.7	-0.4	-0.6	molybdate transporter subunit; periplasmic-binding component of ABC superfamily	modABC	Primary Active Transporters; Metabolism; Building Block Biosynthesis; cofactor, small molecule carrier (Mo); PPi Bond (ATP, GTP, P2) Hydrolysis-driven Active Transporters; ATP-binding Cassette (ABC) Superfamily + ABC-type
b0764	***modB***	0.5	1.2	0.0	-0.1	molybdate transporter subunit; membrane component of ABC superfamily	modABC (modAp1)	Transport; ATP-binding Cassette (ABC) Superfamily + ABC-type Uptake Permeases; Primary Active Transporters; PP_i Bond (ATP, GTP, P2) Hydrolysis-driven Active Transporters; membrane component, cell structure;
b1469	***narU***	-1.3	-4.7	-1.1	-1.9	nitrate/nitrite transporter	narU	Transport; Major Facilitator Superfamily (MFS); N metabolism; Electrochemical potential driven; Porters (Uni-, Sym-, Anti-porters); cell structure; membrane;
b4067	***actP***	5.1	-0.2	-0.1	-0.3	acetate permease	acs-yjcHG (acsp1)	Transport; Electrochemical potential driven transporters; Porters (Uni-, Sym-,Anti-porters); cell structure; membrane
b2092	***gatC***	1.6	-1.7	1.1	0.4	galactitol-specific enzyme IIC component of PTS	gatYZABCD (gatYp)	Transport; (PEP-dependent PTS) C utilization; Group Translocators; Phosphotransferase Systems: PTS Galactitol (Gat) Family, cell structure; membrane; Metabolism;
b2093	***gatB***	1.7	-1.4	1.3	0.7	galactitol-specific enzyme IIB component of PTS	gatYZABCD (gatYp)	Transport; (PEP-dependent PTS) C utilization; Group Translocators; Phosphotransferase Systems: PTS Galactitol (Gat) Family, cell structure; membrane; Metabolism
b2094	***gatA***	2.0	-1.5	1.5	1.0	galactitol-specific enzyme IIA component of PTS	gatYZABCD (gatYp)	Transport; (PEP-dependent PTS) C utilization; Group Translocators; Phospho-transferase Systems: PTS Galactitol (Gat) Family, cell structure; membrane; Metabolism
b3453	***ugpB***	-0.9	1.6	-0.4	-0.4	periplasmic-binding component of an ABC superfamily glycerol-3-phosphate transporter	ugpBAECQ	Transport; Building block biosynthesis; fatty acid/ phosphatidic acid metabolism; C & energy metabolism; aerobic and anaerobic respiration; central intermediary metabolism:

								glycerol.
b4482	***yigE***	-0.1	2.4	-0.5	-0.5	predicted protein	yigE	
b1829	***htpX***	0.1	1.8	0.1	0.0	predicted endopeptidase	htpX	Cell processes; adaptation to thermal stress; cell structure
b3055	***htrG***	-0.4	0.9	-0.5	-0.6	predicted signal transduction protein (SH3 domain)	htrG-cca (htrGp2)	Cell Structure; membrane
b1113	***ycfS***	0.2	1.2	-0.3	-0.6	conserved protein	ycfS	Cell wall remodeling
b1255	***yciC***	0.3	0.8	-0.5	0.0	predicted inner membrane protein	yciC	Cell Structure; membrane
b1473	***yddG***	-0.2	-1.2	-0.2	-0.3	predicted methyl viologen efflux pump	yddG	Cell Structure; membrane
b1806	***yeaY***	0.2	1.5	-0.3	0.0	predicted lipoprotein	yeaZY (yeaZp)	Cell Structure; membrane
b3471	***yhhQ***	0.0	2.3	0.2	0.0	conserved inner membrane protein	yhhQ	Cell Structure; membrane
b3095	***yqjA***	0.0	2.0	-0.3	-0.4	conserved inner membrane protein	yqjAB (yqjAp1)	Cell Structure; membrane
b1080	***flgI***	1.4	-0.5	1.7	0.9	predicted flagellar basal body protein	flgBCDEFGHIJ	Metabolism; Biosynthesis; flagellum; macromolecules (cellular constituent) cell processes; motility (incl. chemotaxis, energytaxis, aerotaxis, redoxtaxis), cell structure;
b1057	***yceJ***	-0.2	0.7	-0.3	-0.4	predicted cytochrome b561	yceJ	Metabolism; macromolecules (cellular constituent) biosynthesis; lg. molecule carriers; cytochromes
b2458	***eutD***	1.3	-0.4	1.0	0.9	predicted phosphotransacetylase subunit	eutDMPQST	Metabolism; C utilization; amines
b0925	***ycbB***	-0.5	1.4	-0.1	-0.2	predicted carboxypeptidase	ycbB	Metabolism; Macromolecule Degradation; proteins/peptides/glycopeptides
b1477	***yddM***	-0.7	-1.9	-0.5	-0.5	predicted DNA-binding transcriptional regulator	yddM	Regulation; Transcriptional Regulation, cell processes; defense/survivial
b3082	***ygjM***	0.2	1.4	-0.1	-0.5	predicted DNA-binding transcriptional regulator	ygjMN	Regulation; Transcriptional Regulation;
b0377	***sbmA***	0.3	1.5	0.2	0.0	predicted transporter	sbmA-yaiW (sbmAp)	Transport; Primary Active Transporters; PP_i bond (ATP, GTP, P2) Hydrolysis-driven Active Transporters;
b0495	***ybbA***	-0.1	1.0	0.1	-0.1	predicted transporter subunit: ATP-binding component of ABC superfamily	ybbA	Transport; ATP-binding Cassette (ABC) Superfamily + ABC-type Uptake Permeases; Primary Active Transporters; PP_i Bond (ATP, GTP, P2) Hydrolysis-driven Active Transporters; ATP binding cytoplasmic component
b0805	***fiu***	-0.1	-1.4	-0.7	0.8	predicted iron outer membrane transporter	fiu	Transport Fe acquisition, cell processes; adaptation to stress; cell structure; membrane
b1902	***yecI***	0.2	2.8	0.6	0.7	predicted ferritin-like protein	ftnB (ftnBp2)	Transport; Fe acquisition; cell processes; adaptation to stress;
b4068	***yjcH***	5.4	-0.2	-0.1	-0.3	conserved inner membrane protein; acetate transport	acs-yjcHG (acsp2)	
b1843	***yobB***	0.0	1.9	-0.1	-0.2	conserved protein	exoX-yobB	
b0378	***yaiW***	0.0	1.2	0.2	0.1	predicted DNA-binding transcriptional regulator	sbmA-yaiW (sbmAp)	
b1056	***yceI***	0.0	1.4	-0.1	-0.5	secreted protein	yceI	
b1254	***yciB***	0.2	0.8	-0.4	-0.1	predicted inner membrane protein	yciB	
b1464	***yddE***	-0.2	-2.5	-0.1	-0.1	conserved protein	yddE	
b1535	***ydeH***	-0.2	2.2	-0.3	-0.6	conserved protein	ydeH	

b1846	***yebE***	0.0	3.0	-0.5	0.0	conserved protein	yebE	
b2602	***yfiL***	-0.8	1.4	-0.9	-0.7	predicted protein	yfiL	
b1452	***yncE***	0.2	-6.0	-0.2	0.2	conserved protein	yncE	
b1436	***yncJ***	0.0	1.6	0.0	0.0	predicted protein	yncJ	
b3096	***yqjB***	-0.3	1.7	-0.5	-0.8	conserved protein	yqjAB (yqjAp1)	
b3097	***yqjC***	-1.8	0.3	-2.0	-2.0	conserved protein	yqjCDEK	
b3098	***yqjD***	-2.0	-0.2	-2.2	-2.0	conserved protein	yqjCDEK	
b3099	***yqjE***	-1.9	-0.3	-2.2	-2.0	conserved inner membrane protein	yqjCDEK	
b3207	***yrbL***	-1.2	0.3	-1.5	-1.0	predicted protein	yrbL	
b4217	***ytfK***	-0.5	0.7	-0.4	-0.4	conserved protein	ytfK	
