# Supplementary material for: E Unibus Plurum: Genomic Analysis of an Experimentally Evolved Polymorphism in Escherichia coli
Source: PLoS Genet. 2009 Nov 6;5(11):e1000713. doi: 10.1371/journal.pgen.1000713 (PMC2763269; doi:10.1371/journal.pgen.1000713)
Supplement: Table S3 — Sequencing primers. (0.13 MB PDF) [file pgen.1000713.s007.pdf]

Supplementary Table 3. Sequencing primers

Name	Sequence (5' →3')
acsseqF	ACCGTTACCGACTCGCATC
acsseqI1	TCGATACCTGGTGGCAGAC
acsseqI2	TGATGTGGTGGCGATTTATATG
acsseqR	GGAGCAGCCGTTTGTTCAG
cyaF1	TCGCCATCAACTTGTCTTTG
cyaI1	GCACTATCACCATCCGCTAA
cyaI2	TGGCAGCTCTACAAGAGTATCG
cyaI3	GTATAACCGCGCGCCAAA
cyaI4	GAAACCGGGCGTTTCAAG
cyaR	CAGGCGGGTGAAACAGTC
glpKf	CGCACGTTTCGGGACTAC
glpKIf	CGGAACCACATACACACCAT
glpKr	CGCTGTAATATGACTACGGGACA
glpRf	AATGACGCGGATCGGCTA
glpRr	GGGTTAGCCGTGGGTTTAG
lamBseqf	TAAGCACCCCACAAAACACA
lamBseqr	CTGCTGATAAACAGAGGACGAT
malTf1	AGGTTTCTGGCCGACCTTAT
malTf2	GAGCTGCCGGAAATCCAC
malTprom	ACAACGTTATCGCTAGTTTGC
malTr1	CGACAGTTCGCTATGGTTGA
malTr2	CGGTGCGGTTTAGTTTGATA
mglDf1	TGATTGCCAGTGCCTTCAC
mglDf2	ATCACATTGTTAAGATACTGTGAAA
mglDI	CCCCAGCAGTTCAACCATC
mglDr	GCTCTGGCGTCAGTTAACTTTG
mlcF1[1]	CTGAATGCTCTCAGGTGAGG
mlcR1[1]	CTCCACCGTTATGCTTCAC
ptaLf	CGGCGGTAACGAAAGAGG
ptaLr	GGCAGTCAGAGATTCGATCC
ptaRf	CCTGCAGAGCTTCAACCTG

ptaRr	AAGGATTAATGCAAATTAAGAGAAT
ptsGLf	CCCGTCTGTTTCACATCGAC
ptsGLr	CAAACGGTACCAGGCAAC
ptsGRf	TCTTTACTGGCGTTGTGCTG
ptsGRr	ACCGGCACGTATCAATTC
rpoSF1[2]	CGGACCTTTTATTGTGCACA
rpoSI[2]	CTGTTAACGGCCGAAGAAGA
rpoSR1[2]	TGATTACCTGAGTGCCTACG
spoTLf	GCCAGGAACAGCAAGAGC
spoTLr	TGCTCTTTATAAGCCCAGTGC
spoTLr2	CCTTCCGGTGTGAAAACGTA
spoTRf	CCAGTACTACCGCACAAATCC
spoTRr	CGCAGATGCGTGCATAAC
