# Supplementary material for: E Unibus Plurum: Genomic Analysis of an Experimentally Evolved Polymorphism in Escherichia coli
Source: PLoS Genet. 2009 Nov 6;5(11):e1000713. doi: 10.1371/journal.pgen.1000713 (PMC2763269; doi:10.1371/journal.pgen.1000713)
Supplement: Table S4 — Failed and low-concentration PCR reactions. (0.15 MB PDF) [file pgen.1000713.s008.pdf]

Supplementary Table 4. Failed and low concentration PCR reactions

ID	gene name	PCR reaction status	gene product
b0012	htgA	bad	heat shock protein HtgA
b0024	yaaY	bad	predicted protein
b0031	dapB	bad	dihydrodipicolinate reductase
b0037	caiC	bad	probable crotonobetaine/carnitine-CoA ligase
b0062	araA	bad	L-arabinose isomerase
b0075	leuL	bad	leu operon leader peptide
b0080	fruR	bad	fructose repressor
b0083	ftsL	bad	cell division protein FtsL
b0089	ftsW	bad	cell division protein FtsW
b0131	panD	bad	aspartate 1-decarboxylase
b0144	yadB	bad	hypothetical protein
b0240	crl	bad	curlin genes transcriptional activator
b0269	yagF	bad	CP4-6 prophage; predicted dehydratase
b0270	yagG	bad	YagG GPH Transporter
b0271	yagH	bad	putative β-xylosidase
b0276	yagJ	bad	CP4-6 prophage; predicted protein
b0304	ykgC	bad	predicted oxidoreductase
b0319	yahE	bad	predicted protein
b0324	yahJ	bad	predicted deaminase
b0335	prpE	bad	predicted propionyl-CoA synthetase
b0349	mhpC	bad	2-hydroxy-6-ketonona-2,4-dienedioate hydrolase
b0375	yaiV	bad	predicted DNA-binding transcriptional regulator
b0400	phoR	bad	phosphate regulon sensor protein PhoR
b0406	tgt	bad	tRNA-guanine transglycosylase
b0437	clpP	bad	ATP-dependent clp protease proteolytic subunit
b0457	ylaB	bad	conserved inner membrane protein
b0460	hha	bad	haemolysin expression modulating protein
b0462	acrB	bad	acriflavin resistance protein B
b0465	o1120	bad	predicted protein
b0466	ybaM	bad	hypothetical protein
b0497	rhsD	bad	RhsD protein precursor
b0517	f349	bad	predicted protein
b0521	ybcF	bad	hypothetical protein
b0525	ppiB	bad	peptidyl-prolyl cis-trans isomerase B
b0564	appY	bad	M5 polypeptide
b0575	ybdE	bad	hypothetical protein
b0586	entF	bad	enterobactin synthetase component F

b0659	ybeY	bad	conserved protein
b0663	b0663	bad	predicted ORF
b0700	rhsC	bad	RhsC protein precursor
b0703	ybfO	bad	conserved protein, rhs-like
b0717	ybgP	bad	putative chaperone
b0779	uvrB	bad	excision nuclease ABC subunit B
b0841	ybjG	bad	undecaprenyl pyrophosphate phosphatase
b0890	ftsK	bad	cell division protein FtsK
b0924	mukB	bad	cell division protein
b0939	ycbR	bad	predicted periplasmic pilin chaperone
b1031	b1031	bad	predicted ORF
b1084	rne	bad	ribonuclease E
b1102	fhuE	bad	outer-membrane receptor for Fe(III)-coprogen
b1117	lolD	bad	outer membrane-specific lipoprotein transporter subunit
b1194	ycgR	bad	protein involved regulation of flagellar motility
b1207	prsA	bad	ribose-phosphate pyrophosphokinase
b1229	tpr	bad	protamine-like protein
b1242	ychE	bad	hypothetical protein
b1250	kch	bad	putative potassium channel protein
b1252	tonB	bad	TonB protein
b1265	trpL	bad	trp operon leader peptide
b1270	btuR	bad	COB(I) alamin adenosyltransferase
b1378	ydbK	bad	putative pyruvate synthase
b1387	maoC	bad	putative ring-cleavage enzyme of phenylacetate degradation
b1409	ynbB	bad	predicted CDP-diglyceride synthase
b1423	ydcJ	bad	conserved protein
b1432	b1432	bad	putative virulence protein
b1461	ydcE	bad	hypothetical protein
b1487	ddpA	bad	subunit of YddO/YddP/YddQ/YddR/YddS ABC transporter
b1489	dos	bad	cAMP phosphodiesterase, heme-regulated
b1495	yddB	bad	predicted ORF
b1496	yddA	bad	hypothetical ABC transporter in gadB 5'region
b1510	ydeK	bad	hypothetical protein in hipA 5'region
b1513	lsrA	bad	fused AI2 transporter subunits of ABC superfamily
b1514	lsrC	bad	LsrC , subunit of LsrA/LsrC/LsrD/LsrB ABC transporter
b1595	ynfL	bad	predicted DNA-binding transcriptional regulator
b1602	pntB	bad	pyridine nucleotide transhydrogenase subunit- beta
b1617	uidA	bad	beta-D-glucuronidase
b1619	hdhA	bad	7-alpha-hydroxysteroid dehydrogenase
b1701	ydiD	bad	short chain acyl-CoA synthetase monomer

b1712	himA	bad	integration host factor alpha-subunit
b1715	pheM	bad	phenylalanyl-tRNA synthetase operon leader peptide
b1770	b1770	bad	predicted DNA-binding transcriptional regulator
b1786	yeaJ	bad	predicted diguanylate cyclase
b1815	yoaD	bad	predicted phosphodiesterase
b1816	yoaE	bad	predicted inner membrane protein
b1831	yebJ	bad	predicted structural transport element
b1837	yebW	bad	predicted protein
b1845	ptrB	bad	protease II
b1859	yebI	bad	hypothetical protein
b1877	yecT	bad	predicted protein
b1903	b1903	bad	phantom gene
b1908	yecA	bad	conserved metal binding protein
b1928	yedD	bad	predicted protein
b1934	yedN	bad	predicted protein, C-ter fragment
b1942	fliJ	bad	flagellar FliJ protein
b1963	yedR	bad	predicted inner membrane protein
b1966	yedS_3	bad	predicted protein, C-ter fragment (pseudogene)
b1976	mtfA	bad	conserved protein
b1978	yeeJ	bad	adhesin
b1997	insC-3	bad	IS2 element protein InsA
b2018	hisL	bad	his operon leader peptide
b2118	yehI	bad	hypothetical protein
b2318	truA	bad	pseudouridylate synthase I
b2360	yfdQ	bad	CPS-53 (KpLE1) prophage; predicted protein
b2420	yfeS	bad	conserved protein
b2432	yfeY	bad	predicted protein
b2457	cchA	bad	predicted protein
b2459	eutT	bad	predicted cobalamine adenosyltransferase
b2463	maeB	bad	NADP-linked malic enzyme
b2500	purN	bad	phosphoribosylglycinamidine myltransferase
b2508	guaB	bad	inosine-5'-monophosphate dehydrogenase
b2520	yfhM	bad	conserved protein
b2535	csiE	bad	stationary phase inducible protein CsiE
b2543	yphA	bad	predicted inner membrane protein
b2557	purL	bad	phosphoribosylformylglycineamide synthetase
b2569	lepA	bad	GTP-binding protein LepA
b2606	rplS	bad	50S ribosomal subunit protein L19
b2647	ypjA	bad	adhesin-like autotransporter
b2751	cysN	bad	ATP sulfurylase (ATP:sulfate adenyltransferase) subunit

b2752	cysD	bad	ATP sulfurylase (ATP:sulfate adenyltransferase)
b2761	ygcB	bad	hypothetical protein in cysH 3' region
b2765	ygcM	bad	6-pyruvoyl tetrahydropterin synthase
b2837	galR	bad	galactose operon repressor
b2843	kduI	bad	5-keto-4-deoxyuronate isomerase
b2852	ygeH	bad	predictedtranscriptional regulator
b2869	ygeV	bad	putative transcriptional regulator
b2920	ygfH	bad	propionyl-CoA:succinate CoA transferase
b3021	ygiT	bad	predicted DNA-binding transcriptional regulator
b3033	yqiB	bad	predicted dehydrogenase
b3038	ygiC	bad	predicted enzyme
b3050	yqiJ	bad	putative oxidoreductase
b3052	rfaE	bad	fused heptose 7-phosphate kinase/heptose 1-phosphate adenyltransferase
b3073	ygjG	bad	probable ornithine aminotransferase
b3102	yqjG	bad	predicted S-transferase
b3125	yhaE	bad	tartronate semialdehyde reductase
b3137	agaY	bad	tagatose-bisphosphate aldolase agaY
b3166	truB	bad	tRNA pseudouridine 55 synthase
b3168	infB	bad	protein chain initiation factor 2
b3181	greA	bad	transcription elongation factor
b3212	gltB	bad	glutamate synthase (NADPH) large chain precursor
b3213	gltD	bad	glutamate synthase (NADPH) small chain
b3220	yhcG	bad	conserved protein
b3230	rpsI	bad	30S ribosomal subunit protein S9
b3297	rpsK	bad	30S ribosomal subunit protein S11
b3310	rplN	bad	50S ribosomal subunit protein L14
b3312	rpmC	bad	50S ribosomal subunit protein L29
b3331	yheI	bad	putative general secretion pathway protein j precursor
b3338	yheB	bad	endochitinase
b3378	yhfU	bad	predicted protein
b3384	trpS	bad	tryptophanyl tRNA synthetase
b3449	ugpQ	bad	glycerophosphoryl diester phosphodiesterase
b3482	rhsB	bad	RhsB core protein with unique extension
b3488	yhiJ	bad	predicted protein
b3489	yhiK	bad	predicted protein
b3521	yhjC	bad	predicted DNA-binding transcriptional regulator
b3593	rhsA	bad	rhsa protein precursor
b3606	yibK	bad	predicted rRNA methylase
b3612	yibO	bad	putative 2,3-bisphosphoglycerate-independent phosphoglycerate
b3643	rph	bad	RNase PH

b3765	yifB	bad	predicted ATP-dependent protease
b3768	ilvG_2	bad	acetolactate synthase II, large subunit, C-ter fragment (pseudogene)
b3772	ilvA	bad	threonine deaminase; threonine dehydratase biosynthetic
b3793	rffT	bad	4-alpha-l-fucosyltransferase
b3826	yigL	bad	sugar phosphatase
b3835	ubiB	bad	2-octaprenylphenol hydroxylase
b3885	yihX	bad	α-D-glucose-1-phosphatase
b3942	katG	bad	catalase hydroperoxidase I
b3955	yijP	bad	conserved inner membrane protein
b4005	purD	bad	phosphoribosylglycineamide synthetase
b4006	purH	bad	phosphoribosylaminoimidazolecarboxamide formyltransferase
b4034	malE	bad	periplasmic maltose-binding protein
b4066	yjcF	bad	conserved protein
b4083	yjcS	bad	predicted alkyl sulfatase
b4099	phnI	bad	phni protein
b4114	yjdB	bad	predicted metal-dependent hydrolase
b4138	dcuA	bad	anaerobic c4-dicarboxylate transporter dcua
b4148	sugE	bad	SugES
b4156	yjeM	bad	YjeM APC transporter
b4177	purA	bad	adenylosuccinate synthetase
b4179	vacB	bad	VacB protein
b4194	yjfT	bad	subunit of L-ascorbate transporting phosphotransferase system
b4199	yjfY	bad	predicted protein
b4200	rpsF	bad	30S ribosomal subunit protein S6
b4201	priB	bad	primosomal replication protein n
b4208	cycA	bad	d-serine/d-alanine/glycine transporter
b4211	ytfG	bad	NAD(P)H:quinone oxidoreductase
b4215	ytfL	bad	predicted protein
b4221	ytfN	bad	conserved protein
b4222	ytfP	bad	conserved protein
b4242	mgtA	bad	Mg(2+) transport ATPase, P-type 1
b4249	yjgI	bad	predicted oxidoreductase
b4256	yjgM	bad	predicted acetyltransferase
b4307	yjhQ	bad	KpLE2 phage-like element; predicted acetyltransferase
b4348	hsdS	bad	type I restriction enzyme ecoki specificity protein (s protei
b0015	dnaJ	low concentration	DnaJ protein
b0495	ybbA	low concentration	hypothetical ABC transporter
b1468	narZ	low concentration	respiratory nitrate reductase 2 alpha chain
b1687	ydiJ	low concentration	predicted FAD-linked oxidoreductase
b1732	katE	low concentration	catalase HPII

b1823	cspC	low concentration	cold shock-like protein CspC
b1916	sdiA	low concentration	sdiA regulatory protein
b2145	yeiS	low concentration	predicted inner membrane protein
b2392	mntH	low concentration	MntH manganese ion NRAMP transporter
b2666	yqaE	low concentration	predicted membrane protein
b2791	truC	low concentration	tRNA pseudouridine 65 synthase
b2866	xdhA	low concentration	xanthine dehydrogenase, molybdenum binding subunit
b3016	ygiQ	low concentration	conserved protein
b4038	yjbI	low concentration	conserved protein
b4058	uvrA	low concentration	excision nuclease
b4068	yjcH	low concentration	conserved inner membrane protein
b4121	yjdF	low concentration	conserved inner membrane protein
b4193	yjfS	low concentration	predicted protein
b4206	ytfB	low concentration	predicted cell envelope opacity-associated protein
