# Supplementary material for: E Unibus Plurum: Genomic Analysis of an Experimentally Evolved Polymorphism in Escherichia coli
Source: PLoS Genet. 2009 Nov 6;5(11):e1000713. doi: 10.1371/journal.pgen.1000713 (PMC2763269; doi:10.1371/journal.pgen.1000713)
Supplement: Table S5 — Primers used for qRT-PCR. (0.12 MB PDF) [file pgen.1000713.s009.pdf]

Supplementary Table 5. Primers used for qRT-PCR

Name	Sequence (5’ →3’)
acsF	TCGTCGCTGCGCATTCT
acsR	CTCGTTGCCGATTTTTTTCC
acs probe	FAM 5’ TTCCGTGGGCGAGCCAATT 3’ BHQ
flgBf	GACGCCTCCTACCGCAGAA
flgBr	CGTTCGCGATCCATATCGA
flgB probe	FAM 5’ ATTCCGGACCAGCCTTCGC 3’ BHQ
lamB probe	FAM 5’ CACAACAGAATGACTGGGAAGCTACCGATC 3’ BHQ
lamBF	CGACACTAACGTGGCCTATTCC
lamBR	GCCATTCGATCAGGTTTTTACC
mdaB probe	FAM 5’ CATGATGTCCGCATCGTTCGCG 3’ BHQ
mdaBF	GCACACTGCGCGACCTT
mdaBR	ACTTCCGCTTTGACATCGTAGTC
